# Decarbonization, Dematerialization, and Economic Gains in Circular Economy for Construction and Demolition Waste Management in Europe

**DOI:** 10.1021/acs.est.6c02401

**Published:** 2026-08-30

**Authors:** Chunbo Zhang, Jinxin Wang, Shuo Wang, Rupert J. Myers, Julia A. Stegemann

**Affiliations:** † Institute of Environmental Planning and Management & Institute of Carbon Neutrality, College of Environmental Science and Engineering, 12476 Tongji University, Shanghai 200092, China; ‡ Department of Civil, Environmental and Geomatic Engineering, 4919 University College London, London WC1E 6BT, U.K.; § Institute of Environmental Sciences, Leiden University, Leiden 2300 RA, Netherlands; ∥ Department of Civil and Environmental Engineering, 4615 Imperial College London, London SW7 2AZ, U.K.

**Keywords:** construction and demolition waste, life cycle assessment, material flow analysis, carbon emissions, material efficiency, value retention, critical raw material

## Abstract

Construction and demolition waste (CDW) offers opportunities for decarbonization, dematerialization, and economic gains through high-value-added circular management. Yet, most of this material is downcycled. This study assesses different CDW circularity strategies in the EU27 + 4 from 2010 to 2050. Results show that continuing current trends in waste management could reduce cumulative carbon emissions by ∼2 Gt CO_2_-eq, material use by ∼24 Gt, and costs by ∼€680 billion compared with virgin material use. Implementing advanced circularity measures could further reduce emissions by 38–48%, material use by 7–8%, and costs by 21%. Germany, the UK, France, and Italy contribute over 70% of these benefits due to their large building stocks. While metals represent only 6% of CDW mass, they drive up to 90% of decarbonization and economic value; minerals enable over 70% of dematerialization across all scenarios. Clean energy transitions may reduce circular economy's carbon advantage as primary production also decarbonizes. Coordinated policies integrating data, technology, incentives, and holistic targets are essential to realize systemic circularity.

## Graphical abstract

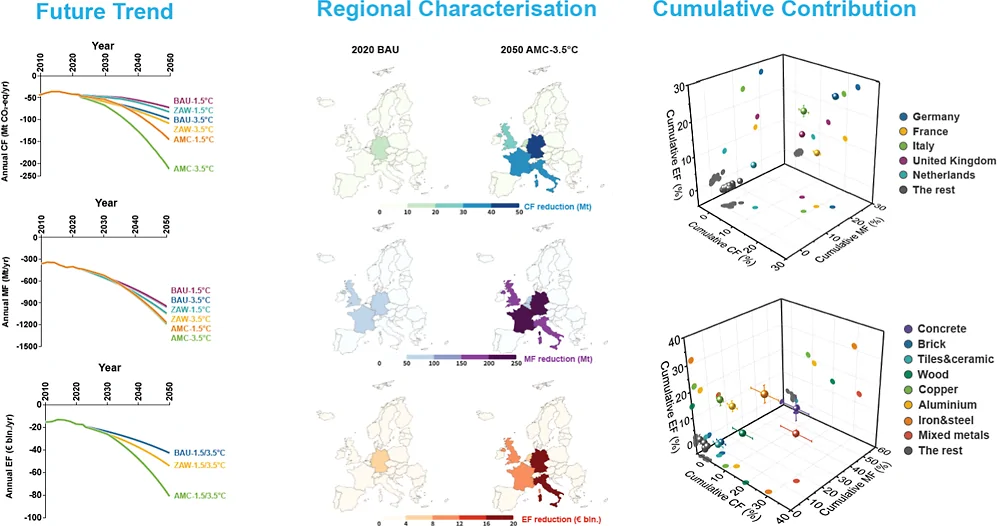

## 1 Introduction

Fast industrialization, population growth, and urbanization during the last several decades have led to unprecedented material extraction and waste generation. Anthropogenic annual material extraction has reached ∼100 gigatonnes (Gt)/y in 2024 and is projected to exceed 150 Gt by 2050.^1^ This surge in resource consumption has also led to a dramatic increase in waste generation, with the release of ∼20 Gt/y of liquid and solid waste in 2021.^2^ The built environment is the biggest material consumer and waste producer, responsible for ∼50% of raw material use and ∼40% of waste generation worldwide.^3^ Raw material use and generation of construction and demolition waste (CDW) from buildings are expected to increase 5-fold and 9-fold, respectively, by 2050 compared to 1970 levels.^1^ Accelerating resource exploitation and waste generation in the built environment creates an urgent need to transform the current patterns of material use and disposal.

The circular economy, which seeks to establish an economically beneficial closed-loop material supply, has become a central focus of global policymaking in response to more environmentally sustainable economic growth and supply chain security.^4^ CDW accounts for over one-third of all waste generated in the EU^5^ and is therefore regarded as a priority target for circularity strategies. The EU Waste Framework Directive (WFD) set a target of 70% CDW recovery by 2020,^6^ and the EU has since emerged as a frontrunner in CDW management, achieving an 88% recovery rate for mineral fractions in that year.^7^ In practice, however, mineral CDW is predominantly downcycled for use as road backfill in Europe.^8^ This pathway faces critical limitations: while motorway construction in the 27 countries of the European Union (EU27) expanded by ∼16,000 km between 2000 and 2010, the subsequent decade added only ∼7000 km—less than half the previous rate—indicating that the road sector can no longer absorb CDW at historical levels.^9^ Moreover, backfilling represents a low-efficiency use of materials and runs counter to the high-value-added recovery pathways promoted in the EU’s new Circular Economy Action Plan.^10^

One of the most prominent merits of the circular economy is its focus on dematerialization—a decrease in the mass of materials used to fulfill societal functions.^11^ Essentially, dematerialization refers to a mode of growth where economic development is decoupled from material requirements.^12^ Nevertheless, a shift toward a dematerialized society may also bring about rebound effects—introducing new challenges while addressing existing ones.^13^ For instance, secondary material production may lead to additional transport requirements, environmental impacts, and energy use. Using new technologies, especially renewable energy, for material recovery may increase the demand for critical and strategic minerals that have much greater embodied material consumption than fossil materials.^1^ Moreover, an increase in greenhouse gas (GHG) emissions represents another major concern.^1,14–17^

In 2015, the United Nations announced the Paris Agreement to address global climate change.^18^ In response, countries have committed to developing initiatives to achieve carbon neutrality.^19^ According to the Intergovernmental Panel on Climate Change (IPCC), waste management accounts for ∼4% of global anthropogenic GHG emissions.^20^ While this share is relatively small, waste recovery can potentially contribute to more embodied carbon emission avoidance—an aspect not yet fully reflected in the IPCC emission accounting framework. Material production accounts for approximately 15–23% of global GHG emissions, with the construction material sector responsible for about two-fifths of these emissions.^21^ It is estimated that effective circular economy practices could reduce global emissions by up to 25%,^22^ underscoring the importance of circularity interventions to climate change mitigation.^23^ More importantly, value retention from waste has gained noticeable momentum recently. An initial assessment reveals that linear material use results in an annual economic loss—termed the “value gap”—of ∼€25 trillion, equivalent to nearly 31% of global gross domestic product (GDP).^24^ This loss could be avoided through circular waste management practices.

Despite the recognition of the potential for circular CDW management to reduce material use, material-related GHG emissions, and economic loss, several critical research gaps remain. First, although studies^25–30^ have shown that circular and material-efficient strategies can reduce GHG emissions from construction material production at scale, it remains unclear whether transitioning to a system in which materials are circulated at high value can further enhance decarbonization. Comparisons between different technical routes for CDW recovery have been explored,^31–35^ but these largely remain at the product level or focus on single CDW fractions. Moreover, except for a few studies,^9,31,36^ additional material inputs required to support CDW recovery processes have not been adequately addressed. Finally, although the environmental and resource benefits of CDW recovery are increasingly recognized, the economic implications of implementing advanced circularity systems at scale remain poorly understood. These limitations hinder policymakers, industry stakeholders, and researchers from fully assessing the climate change mitigation, resource efficiency, and economic viability of circularity strategies in CDW management. Therefore, a critical unanswered question is: Can a systemic shift toward a value-retention-based CDW circularity achieve simultaneous and scalable gains in decarbonization, dematerialization, and economic performance across Europe?

This research aims to address existing gaps in decarbonization, dematerialization, and economic viability within the context of advancing circular CDW management across 31 European countries—the EU27 and the United Kingdom (UK), Norway, Iceland, and Switzerland—from 2010 to 2050. This study focuses on CDW from buildings rather than infrastructure, as buildings are the primary focus of EU circular economy policies.^10^ The diversity of building structures and material composition offers substantially higher potential for high-value material circulation (e.g., reuse and closed-loop recycling). Infrastructure waste, in contrast, follows largely fixed treatment routes (e.g., on-site asphalt reuse, roadbed backfilling) that leave little room for advanced circularity strategies. Life cycle-based carbon, material, and economic footprint (EF) assessments are used to measure the extent of decarbonization, dematerialization, and the economic costs associated with implemented circularity strategies. Three foreground waste management scenarios are used in this evaluation: (i) business-as-usual (BAU), which continues current CDW management practices; (ii) zero avoidable waste (ZAW), which aims for near-full recovery of all CDW, except for minor unavoidable fractions; and (iii) advanced material circulation (AMC), which not only seeks full recovery but also emphasizes high-quality treatment operations such as reuse and recycling to maximize the value of CDW. As decarbonization is highly associated with the adoption of renewable energy,^37^ two background energy transition scenarios are established based on the integrated assessment model IMAGE 3.2.^38^ These two scenarios represent moderate and ambitious clean energy penetration, corresponding to global temperature increases of 3.5 and 1.5 °C, respectively. The findings of this study provide insights into trade-offs and synergies between decarbonization, dematerialization, and economic viability in adopting a circular economy for sustainable CDW management.

## 2 Methods

### 2.1 Overall Approach

The geographical scope of this study covers Europe, including the EU27 and four non-EU countries: the UK, Norway, Iceland, and Switzerland. The temporal scope extends from 2010 to 2050, with 2010–2022 representing historical trends and 2023–2050 reflecting the projection period for future trends. The methodological framework of this study consists of three main modules: (i) characterizing waste generation in each country, (ii) defining waste management and energy transition scenarios, and (iii) quantifying the levels of dematerialization and decarbonization in each scenario. This study employs a generation-based waste accounting system, and therefore, the supply and trade of materials across countries are not considered. Three waste management scenarios—BAU, ZAW, and AMC—are established to provide a baseline and two more advanced systems for waste management. Additionally, two energy transition pathways—associated with temperature change limits of 3.5 and 1.5 °C—are established to evaluate how clean energy would contribute to decarbonization and dematerialization in CDW management. Life cycle-based footprint analysis is employed to evaluate the extent of decarbonization, dematerialization, and economic gains, by calculating material footprint (MF) (in kg of total material requirements), carbon footprint (CF) (in kg of carbon dioxide equivalents, kg CO_2_-eq), and EF (in € of financial cost), respectively.

### 2.2 Waste Generation Estimation

#### 2.2.1 Preliminary Data for Total CDW Generation

Eurostat reports waste generated by European countries based on the European Waste Classification for Statistics (EWC-Stat)^39^ and Statistical Classification of Economic Activities (NACE).^40^ Overall CDW generation for each country is estimated by including waste categories W061 (metal wastes, ferrous), W062 (metal wastes, nonferrous), W063 (metal wastes, mixed ferrous and nonferrous), W071 (glass wastes), W074 (plastic wastes), W075 (wood wastes), W077 (waste containing PCB), and W12B (other mineral wastes, W122 + W123 + W125) from the NACE construction sector (F), in addition to W121 (mineral waste from construction and demolition) across all NACE activities plus households.^41^ As Eurostat only reports waste data biennially (i.e., 2010, 2012, 2014, 2016, 2018, 2020, and 2022), the data for the missing years are extrapolated based on the average of the previous year and the next year. Data for Switzerland are not available from Eurostat and were estimated based on the EU-level waste generation per capita and its population.

It is noted that Eurostat data may both overestimate and underestimate actual CDW generation. Although excavated soil (W126) is excluded from the analytical scope, it can be commingled with mineral CDW and inadvertently accounted for under codes W12B and W121. In Finland, for example, the Valtion Hallinnon Tietojärjestelmä (VAHTI)—the Finnish Environmental Administration’s Register for compliance monitoring data—reported CDW generation of 16.0 megatonnes (Mt) in Finland in 2012, which aligns closely with raw Eurostat data (16.2 Mt).^42^ However, it is estimated that 87.5% of this CDW consists of excavated soil.^42^ This indicates that waste statistics offer a loophole for mixing soil into CDW, thereby distorting the representation of actual CDW generated from construction and demolition activities. Such distortion is likely not unique to Finland. To maintain consistency in Eurostat data across countries, mixed soil is retained in the reported waste figures. Conversely, Eurostat may underreport actual CDW generation due to unrecorded flows from on-site recovery and reuse, exemptions, and illegal activities.^43^ These missing amounts are also not accounted for in this study. Details are provided in Text S1.1 of the Supporting Information.

#### 2.2.2 Approximation of CDW from Infrastructure

The CDW figures from Eurostat include waste generated from the construction, demolition, and refurbishment of nonbuilding infrastructure (e.g., roads, track rails, tunnels, and bridges). Since Eurostat does not discriminate between different sources of the reported CDW, those originating from infrastructure are estimated and removed from the total CDW arising. The study shows that typical materials used in infrastructure include minerals (e.g., aggregate, concrete, stone, asphalt, bricks), metals (e.g., aluminum, zinc, copper, steel), and wood.^44^ Among them, aggregate, asphalt admixture fractions, and concrete account for more than 90% of material use for European infrastructure.^44^ Given their dominance, these three nonbuilding-derived material streams are quantified in this study; other fractions (metals, wood, etc.) are excluded due to their minor mass contribution.

Aggregate is the main raw material for roads, accounting for ∼60% of all materials used for infrastructure in Europe.^44^ Eurostat does not have data in particular on waste road aggregate. Moreover, road resurfacing will not disturb the aggregate embedded in the road base and subbase,^45^ and any waste road aggregate generated may go unrecorded if it is reused in situ for road engineering.^43^ These reasons render waste road aggregate generation a “black box” for waste statistics. Although not directly reported, railway track ballast is captured under the European List of Waste (LoW) code 17 05 07/08 in some national statistics, including those of England,^46^ France,^47^ Denmark,^48^ and Spain.^49^ The average share of waste track ballast in total CDW in these four countries (1.08%) was used to extrapolate the amount generated in other countries. Malta, Iceland, and Cyprus have no operational railways and are therefore assigned zero waste track ballast.^50^ Details are provided in Text S1.2 of the Supporting Information.

Asphalt makes up ∼30% of all materials used for infrastructure in Europe.^44^ The three major types of asphalt products are asphalt-based paints, roofing asphalts, and paving asphalts. The European Asphalt Pavement Association (EAPA) provides statistics for the production and reclamation of asphalt waste in some European countries.^51^ Asphalt-based roofing materials can be recycled into paving asphalt by existing techniques,^52^ which makes it hard to separate roofing asphalt from paving asphalt waste. As asphalt-based paints and asphalt roofing materials represent a small fraction of asphalt compared to paving asphalt,^52^ it is assumed that all asphalt waste is from the road sector. Based on data from EAPA, asphalt waste is estimated to account for from 5% in Hungary and Iceland to 48% in Sweden. For countries with available data but missing years, missing values were extrapolated using the country’s average share between 2013 and 2022. For countries with no data reported, an average value of 20% is assumed. Details are provided in Text S1.2 of the Supporting Information.

Waste concrete arises from both the infrastructure and building activities. In the infrastructure sector, concrete is used in bridges, railways, and tunnels.^44^ However, the data for waste concrete from infrastructure are not available and only account for a minor fraction of total CDW generated from infrastructure (less than 10% in Europe^44^). Therefore, this study assumes that all waste concrete originates from buildings.

#### 2.2.3 Characterization of Waste Fractions

Before further characterizing the waste fractions into more detailed subcategories, CDW from infrastructure is subtracted from the total CDW generation. The estimated amount of track ballast and asphalt is removed from the mineral CDW under EWC-Stat codes W121 and W12B. Then, waste generation data under the EWC-Stat codes are converted into LoW codes. The 51 waste categories in the EWC-Stat are aggregations of the 824 origin-based waste codes listed in the LoW.^53^ The LoW organizes waste into 20 chapters based on their industries of origin, with CDW categorized under Chapter 17. As the LoW system provides a more detailed and sector-specific description of CDW, data for overall CDW generation for each country are converted to the LoW codes based on guidance provided by the EC^54^ and Eurostat.^55^ In particular, the composition of mineral CDW (W121 and W12B) is broken down into concrete, brick, ceramics, gypsum, and insulation based on the report;^54^ nonferrous CDW (W062) is broken down into aluminum and copper with the share 58:42, based on the national waste statistics of Denmark,^48^ France,^47^ Spain,^49^ and the UK.^46^ Mixed metals (W063) are not disaggregated, but their footprint is calculated based on the share of iron and steel, aluminum, and copper by weight—90:6:4. Details regarding this conversion are given in Text S1.3 of the Supporting Information.

#### 2.2.4 Future CDW Generation Projection

JRC reported the future trends for CDW generation in Western Europe, Southern Europe, Northern Europe, and Eastern and Central Europe until 2050.^54^ For each investigated country, the projected generation of each CDW fraction was derived by applying the region-specific relative growth factors (Table S1) reported by the JRC to the national CDW generation level in the base year (2020). Details are provided in Text S1.4 of the Supporting Information.

### 2.3 Scenario Development

Three waste management scenarios are developed: BAU, ZAW, and AMC, representing varying levels of ambition in CDW management, from no additional effort to significant advancements.

The BAU scenario assumes that each country will maintain its current CDW management practices, many of which were implemented to achieve the WFD target for 70% CDW recovery,^6^ as of 2020. Data on the status quo of CDW management in each country were collected from a Eurostat waste treatment data set^56^ that uses Recovery and Disposal (R&D) codes^6^—to categorize different recovery and disposal processes, e.g., recycling, backfilling, landfill, and energy recovery.

The EU has not yet established a target beyond the 2020 70% CDW recovery goal, but some countries have already set more ambitious goals, such as the UK’s “Zero Avoidable Waste by 2050” target^57^ and “Full Circularity by 2050” goal of The Netherlands.^3^ The ZAW scenario assumes achievement of a near 100% recovery rate by eliminating the disposal of avoidable waste (e.g., through landfill and incineration) by 2050.

The 70% CDW recovery target may have effectively enhanced the recovery rate in the EU but falls short of ensuring resource efficiency and environmental sustainability in waste management^58^ since most mineral CDW has been downcycled as backfill or road aggregate in Europe.^7^ The WFD also introduced the Waste Hierarchy, a framework used to prioritize waste management practices from the most preferred option—prevention—to the least preferred—disposal.^6^ In accordance with this framework, the EU should maximize the value embedded in CDW by encouraging reuse and recycling.^10^ The AMC scenario aims not only for a 100% recovery rate but also emphasizes advanced waste management practices that adhere to the Waste Hierarchy,^6,59^ including reuse, upcycling and closed-loop recycling that retain material value, rather than downcycling, energy recovery, or disposal (e.g., landfill and incineration). To operationalize this ambition, the AMC scenario sets specific material-dependent targets for reuse, upcycling, and recycling, which gradually increase to their maximum achievable levels by 2050. The maximal reuse, upcycling, and recycling potential of each material is derived from the report of ref 54 and Zhang et al.^9^ (Table S2). The assumptions and references for the three scenarios are detailed in Text S2 of the Supporting Information.

Clean energy can significantly impact both material use and GHG emissions within a technological system for waste processing and treatment. The energy transition scenarios in this study are developed based on the integrated assessment model IMAGE, developed by the PBL Netherlands Environmental Assessment Agency. This model integrates a variety of disciplines, including economics, energy, land use, and climate science, to provide comprehensive insights into future environmental and policy scenarios.^38^ IMAGE 3.2 creates various socioeconomic transition scenarios by combining Shared Socioeconomic Pathways (SSPs)^60^ with Representative Concentration Pathways (RCPs).^61^ For this study, the SSP2 scenario, known as the “middle-of-the-road” pathway, is employed. The SSP2 predicts moderate population and GDP growth consistent with historical trends. This study examines the IMAGE SSP2-RCP6 and IMAGE SSP2-RCP19 scenarios, which are designed to limit global temperature increases to 3.5 and 1.5 °C, respectively, by the end of the century compared to preindustrial levels.

### 2.4 Footprint Quantification

The Europe-wide carbon, material, and economic footprints of CDW management are quantified according to the following three steps: inventory modeling, impact assessment, and upscaling. The inventory data for modeling waste treatment is mainly collected based on a previous study by the lead author^9^ and is complemented by additional sources of data (Tables S3 and S4).

Waste recovery processes, such as recycling and downcycling, are inherently multifunctional because they simultaneously provide waste treatment and generate secondary materials that displace primary material production. To address this multifunctionality, a substitution approach is applied in the life cycle inventory modeling.^62^ Under this approach, the environmental burdens associated with the production and transport of the displaced virgin materials are credited to the system and subtracted from the impacts of waste treatment. The resulting net carbon and material footprints, therefore, reflect both the impacts of recovery operations and the benefits of avoided primary production. Detailed life cycle inventory data are provided in Text S3 of the Supporting Information.

The decarbonization of waste management is quantified as a life cycle-based CF, expressed in terms of global warming potential over 100 years (in kg CO_2_-eq), as per the IPCC 2021 impact method.^63^ The measurement of dematerialization, on the other hand, is more diverse, involving both mass-based and impact-based indicators,^64^ as well as relative and absolute values.^11^ The life cycle-based MF is used to assess the dematerialization based on the total material requirement (in kg) method.^65^ The life cycle-based EF is quantified in euros (€). All cumulative cost results are discounted using the interest rates for the cost of borrowing for corporations from the European Central Bank.^66^ Finally, the unitary footprint indicator is upscaled by using the disaggregated CDW flows and fates in each country.

## 3 Results

### 3.1 Mineral-Dominated CDW Growth Led by Western Europe

Figure 1 presents the historic (2010–2022) and projected (2023–2050) CDW generation from the building sector across 31 European countries. Annual CDW generation fluctuated between 2010 and 2022, with two distinct downturns likely resulting from the 2008 financial crisis and COVID-19 lockdowns. This is followed by a steady increase from 347 Mt in 2020 to 837 Mt by 2050—a nearly 3-fold rise. As shown in Figure 1a, Germany (23%), the UK (17%), France (16%), and Italy (13%) collectively account for 69% of cumulative CDW generation from 2010 to 2050. These four nations, mostly located in Western Europe, represent the continent’s largest economies.

**Figure 1:**
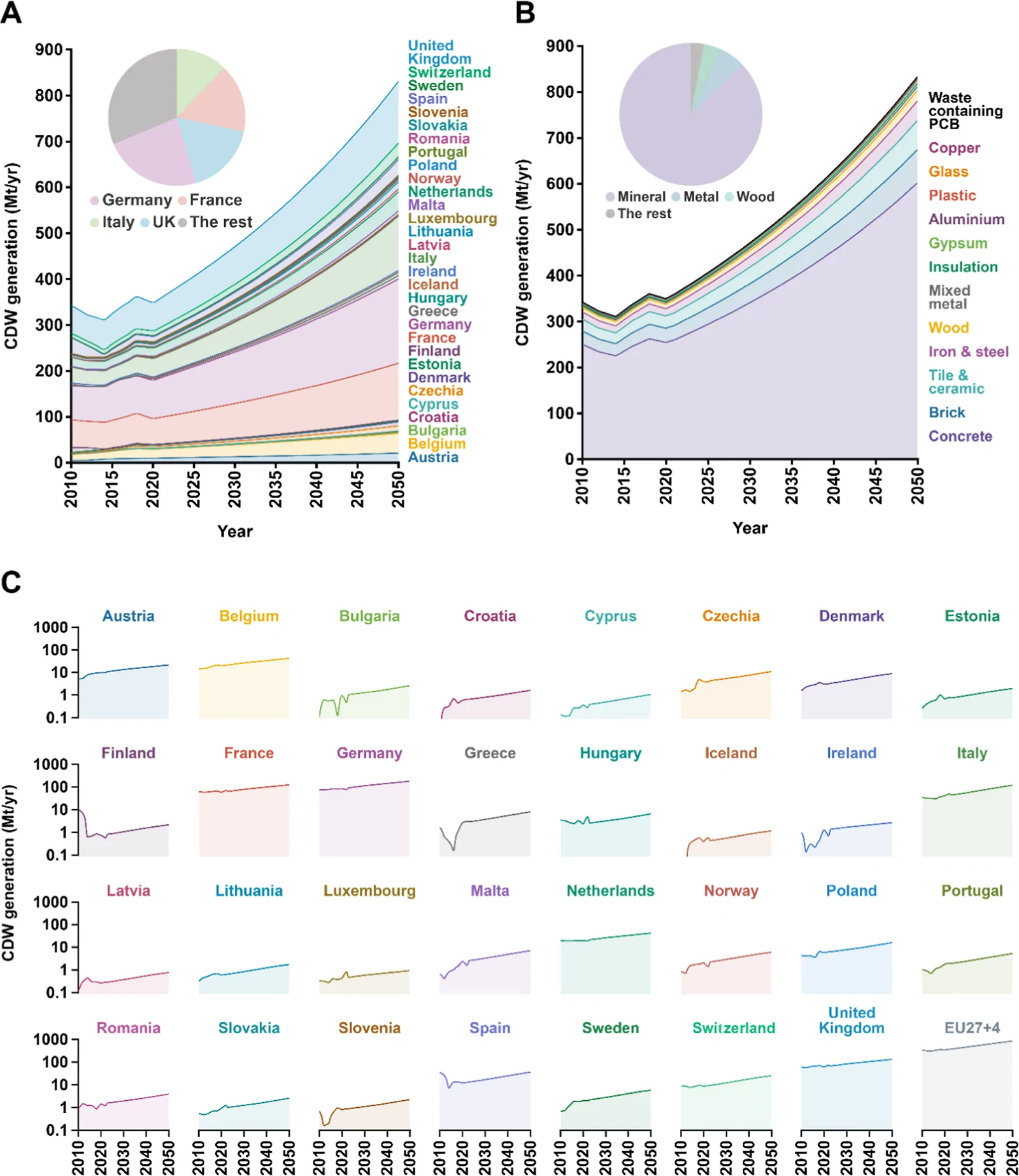
Annual CDW generation in Europe from 2010 to 2050. (A) Country-based breakdown of CDW generation. (B) Material-based breakdown of CDW generation. (C) CDW generation in each country. Note: The pie charts in panels (A,B) represent the contribution of cumulative CDW generation from 2010 to 2050.

Figure 1c illustrates the trends for CDW generation from the building sector across individual countries over time. Historically, larger economies exhibit relatively stable trajectories, whereas smaller countries—such as Bulgaria, Greece, Ireland, and Slovenia—display greater volatility, including a noticeable decline after 2010, followed by recovery. Finland represents a unique case, with CDW generation peaking in 2010 and stabilizing around 2014, diverging from the more stable patterns of its Nordic neighbors, Sweden and Norway. As explained earlier in Section 2.2, this trend may result from the inclusion of soil between 2010 and 2014. However, other mechanisms are also plausible: the 2008–2010 construction boom followed by a postcrisis collapse; a one-off demolition wave of Soviet-era panel buildings; and the effect of the 2014 revision to the Finnish Waste Act.

The growth in CDW generation is predominantly driven by mineral waste (as shown in Figure 1b), which constitutes approximately 90% of the cumulative CDW generated within the time span 2010–2050. Metals and wood account for notably smaller shares, at about 6% and 3%, respectively. Among mineral fractions, concrete is the largest contributor, representing nearly 73% of all CDW, followed by brick (9%) and tile and ceramic (8%). Other minerals, such as gypsum and insulation, make up negligible proportions, each contributing less than 1%. In general, projections exhibit smoother temporal trends, highlighting that real-world dynamics—such as macroeconomic shocks (e.g., the aforementioned financial crises and pandemics) and episodic events, including the treatment of historical stockpiles or concentrated demolition activities—introduce variability that extends beyond what modeling approaches can capture.

### 3.2 Zero-Waste Yields Marginal Gains for CDW Management in Europe

Figure 2A illustrates the cumulative amount of CDW managed from 2010 to 2050 under each of the BAU, ZAW, and AMC scenarios. Figure 2B shows the share of CDWs undergoing each technological management route.

**Figure 2:**
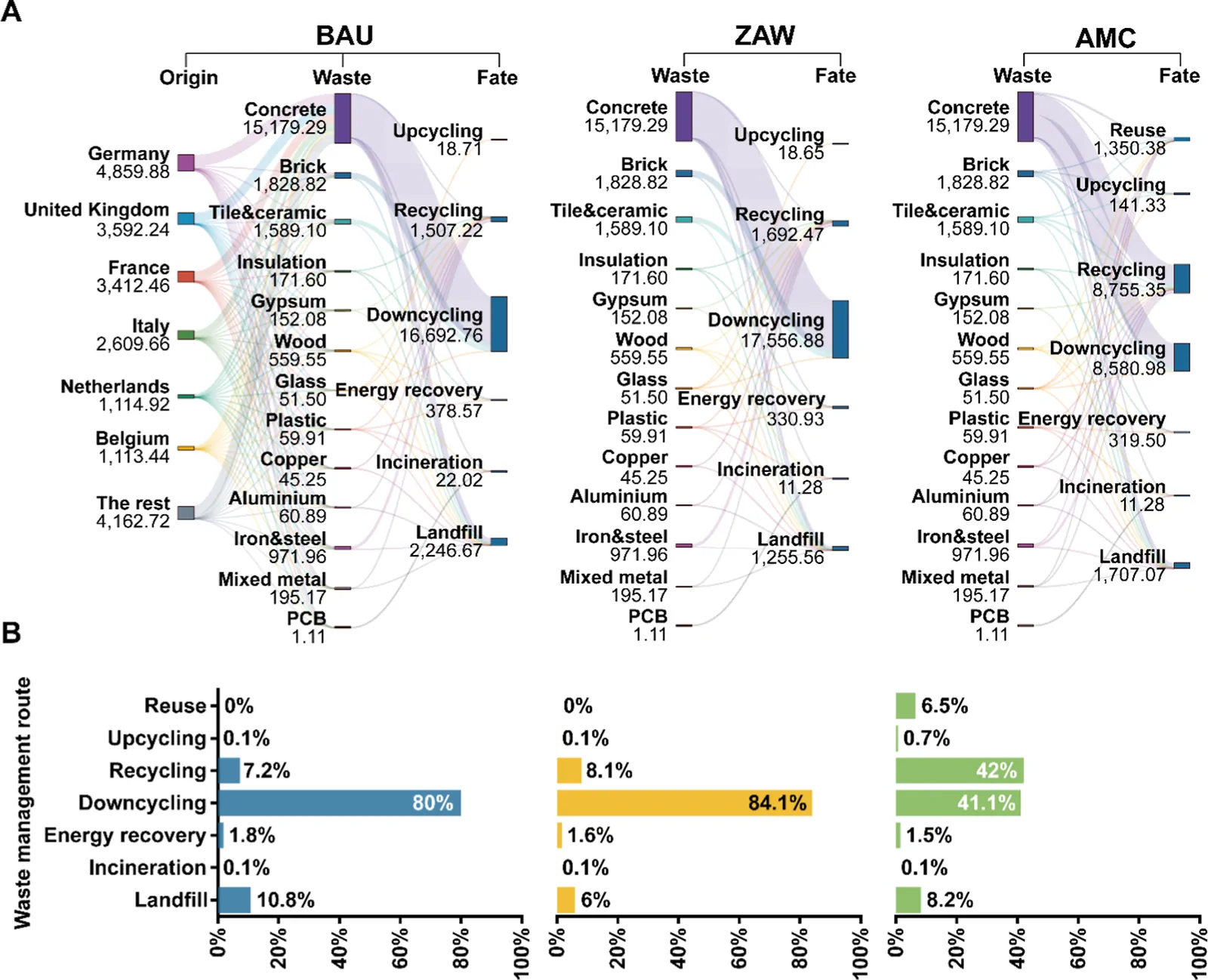
Cumulative quantity of CDW managed from 2010 to 2050 in Europe (EU27 + 4) by country, material, and waste management route (unit: Mt). (A) Sankey diagrams of CDW management in three scenarios. (B) Contribution of each waste management route in CDW management in three scenarios. BAU: business-as-usual, ZAW: zero avoidable waste, AMC: advanced material circulation, and PCB: CDW containing polychlorinated biphenyls.

Under the BAU scenario, which extends current management practices to 2050, approximately 80% of CDW is downcycled—about 90% of which consists of concrete downcycling. The recycling rate—defined here as closed-loop recycling and excluding upcycling and downcycling—remains low at 7%, while 11% of CDW is still landfilled.

The ZAW scenario phases out landfilling and incineration of avoidable CDW by 2050, yet results remain similar to BAU. Due to Europe’s already high recovery rates, pursuing full circularity in ZAW yields only marginal gains: recycling increases from 7% to 8%, and downcycling from 80% to 84%.

In contrast, the AMC scenario goes beyond waste recovery to prioritize the circulation of materials in the economy at high value. Reuse accounts for 7% and upcycling for 1%, while the recycling rate rises substantially to 42%—compared to below 10% in both BAU and ZAW. This demonstrates that policies focused solely on “zero waste” or “full circularity” are inadequate to transform CDW management; meaningful improvement requires targeted strategies that promote high-value material recycling and reuse.

### 3.3 Energy Transition May Reduce the Climatic Advantages of the Circular Economy

The carbon, material, and economic footprints of CDW management are quantified based on the quantities of CDW processed through various technological routes and the associated life cycle footprint indicators. Figure 3A shows that current circular economy practices in Europe’s CDW management sector have already begun to yield net benefits in GHG emissions and material reduction and cost savings. Figure 3B compares the projected reduction of carbon, material, and economic footprints in the AMC-3.5 scenario in 2050 (best practice) with that of the BAU scenario (baseline) in 2020 across individual countries (Figure 3B).

**Figure 3:**
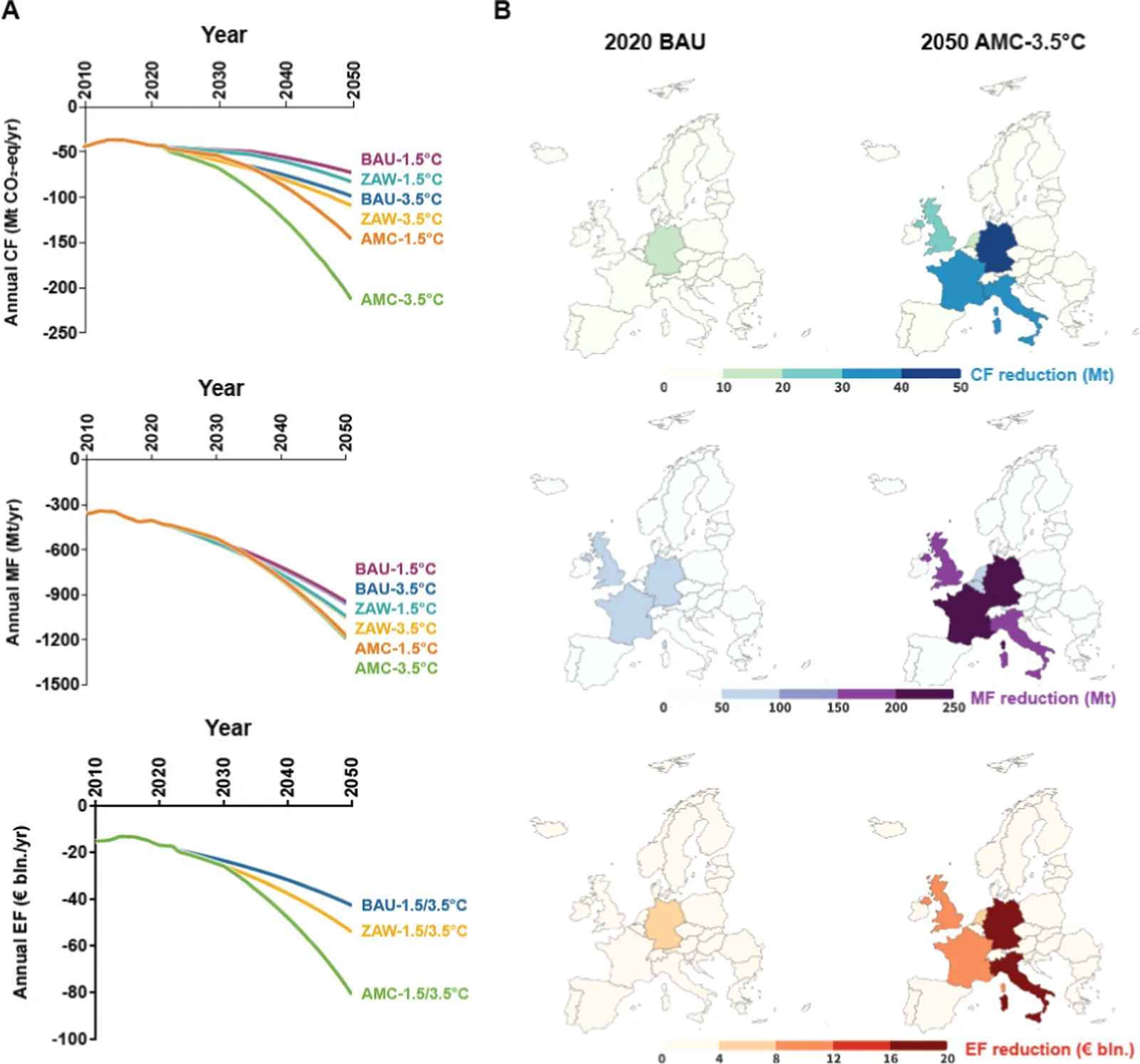
Per-annum CF, MF, and EF of CDW management in Europe under six scenarios from 2010 to 2050. (A) Annual CF, MF, and EF of CDW management in EU27 + 4. (B) Comparison of CF, MF, and EF reduction in the AMC-3.5 °C scenario in 2050 compared to the 2020 levels in each country. BAU: business-as-usual scenario, ZAW: zero avoidable waste scenario, AMC: advanced material circulation, 3.5 °C: IMAGE SSP2-RCP6, and 1.5 °C: IMAGE SSP2-RCP19.

In 2020, current CDW management contributed to 41 Mt CO_2_-eq/y of GHG emission reduction in Europe compared to using virgin material. All six scenarios exhibit increasing trends in CF reduction after 2020 (Figure 3A). A reduction of 98 Mt CO_2_-eq/y of GHGs can be expected in 2050 in the BAU-3.5 °C scenario, representing a more than 2-fold reduction in CF compared to 2020 levels. The ZAW-3.5 °C scenario could reduce GHG emissions by a further 10 Mt CO_2_-eq, achieving a GHG reduction of 108 Mt CO_2_-eq/y in 2050. The AMC-3.5 °C scenario shows a more considerable GHG reduction of 212 Mt CO_2_-eq/y by 2050. An advanced material recovery pathway, therefore, clearly yields GHG emission reduction.

Interestingly, Figure 3A reveals that a transition to renewable energy systems would decrease the potential to reduce GHG emissions across all waste management scenarios. Under a 1.5 °C-aligned energy transition pathway, CDW management in the BAU, ZAW, and AMC scenarios is projected to reduce approximately 72, 82, and 145 Mt CO_2_-eq/y, respectively, in 2050 compared to using virgin materials. These values represent 26–67 Mt CO_2_-eq less GHG emissions reduction compared to their counterparts under a 3.5 °C pathway. This suggests that the increasing deployment of cleaner energy and low-carbon technologies—such as renewable electricity and carbon capture and storage (CCS)—in construction material industries may narrow the emission gap between primary and secondary raw material (2RM) production, rather than reversing their relative carbon intensities. While similar decarbonization measures can also be applied to 2RM recovery processes, reductions in the carbon intensity of primary production will diminish the relative GHG savings achievable through recycling. Yet, it is worth noting that the narrowing carbon advantage of circularity under cleaner energy scenarios is partly a methodological consequence of the substitution method. Zhang et al.^36^ examined different multifunctionality modeling approaches—system expansion, substitution, and allocation—in the context of concrete recycling and found that they would not reverse the overall conclusions. However, given the diversity of CDW materials and treatment routes, alternative allocation approaches would likely yield different quantitative results.

As might be expected, MF reductions are predominantly driven by circularity strategies, with energy transitions only playing a minor role. In 2020, CDW circulation practices in Europe decreased the MF by 405 Mt/y compared to using virgin materials (Figure 3A). By 2050, the BAU scenario is projected to reduce the MF by 946–958 Mt/y, depending on energy transition scenarios. Both the ZAW and AMC scenarios offer further MF reductions, by 1042–1054 Mt/y and 1171–1191 Mt/y, respectively, depending on energy transition scenarios. A more ambitious energy transition slightly increases the MF across all waste management scenarios, indicating that clean energy adoption may impose modest additional material demands—in material recovery—revealing a latent trade-off between fossil-fuel-based materials and energy transition minerals.^1^

The EF demonstrates a trend consistent with both the carbon and material footprints. In 2020, the estimated total economic gains reached approximately €17 billion/y in the investigated regions. By 2050, these gains are projected to rise to around €43 billion/y under the BAU scenario, €54 billion/y under the ZAW scenario, and €81 billion/y under the AMC scenario. Critically, these results reveal that the economic benefits of circularity are substantial and escalating—making the business and policy case for accelerated action clear, as circular strategies deliver decarbonization, material efficiency, and rising economic returns in tandem.

Figure 4A,B shows the cumulative reductions of carbon, material, and economic footprints across various scenarios from 2010 to 2050. The BAU and ZAW scenarios are projected to reduce the cumulative CF by approximately 2.0–2.6 Gt. The AMC scenario offers a greater GHG emission reduction, ranging from 2.8 to 3.6 Gt CO_2_-eq, depending on energy transition scenarios. The BAU scenario is expected to reduce about 24 Gt of material use, with the ZAW scenario achieving a slightly higher reduction of 25 Gt. The AMC scenarios deliver the highest MF reduction, of 26 Gt, but the difference among the three scenarios is probably not practically significant.

**Figure 4:**
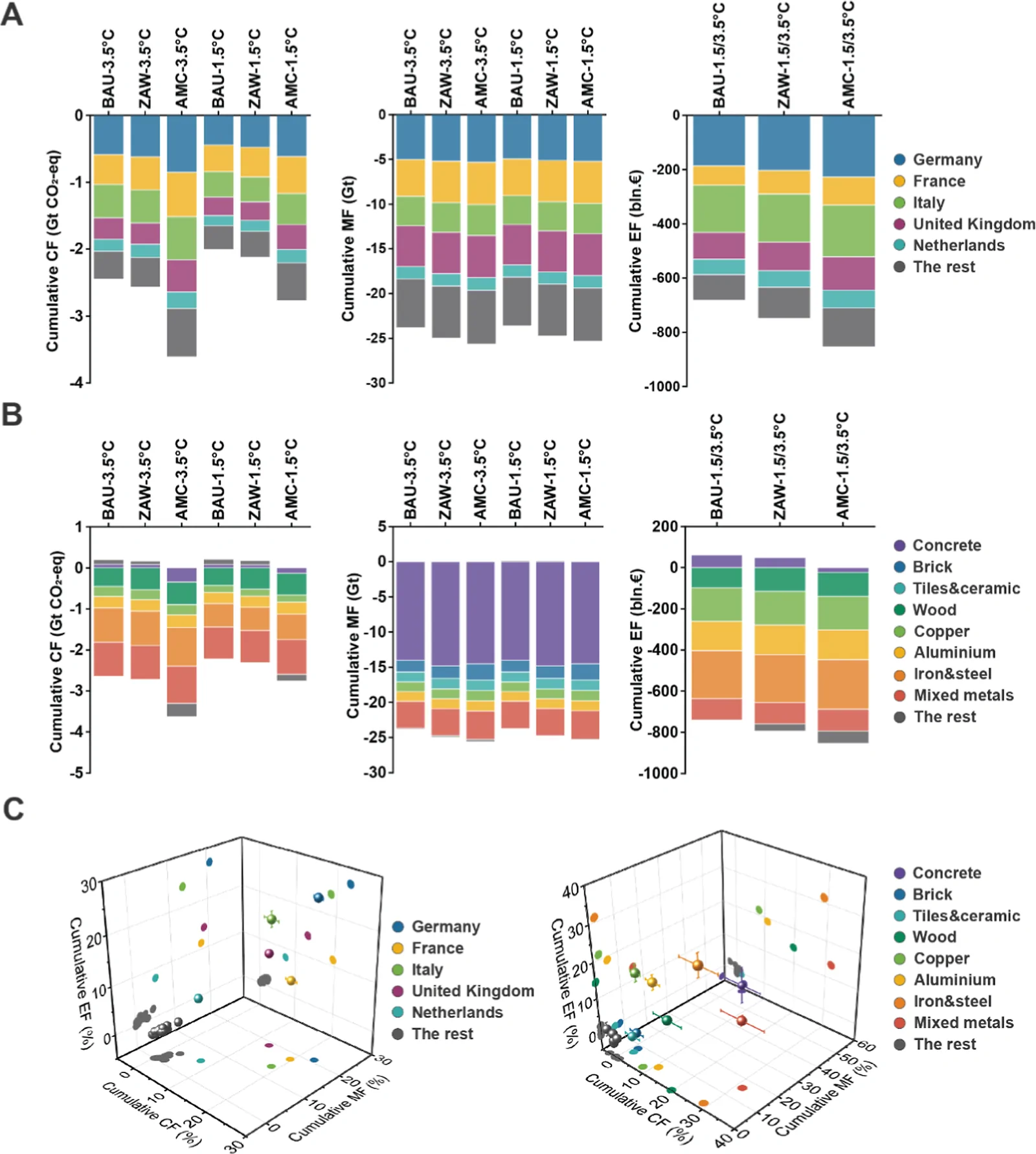
Cumulative CF, MF, and EF of CDW management in Europe (EU27 + 4) under six scenarios from 2010 to 2050. (A) Breakdown of cumulative CF, MF, and EF by countries. (B) Breakdown of cumulative CF, MF, and EF by material categories. (C) Contribution analysis of cumulative CF, MF, and EF; values below zero on the axes indicate an increase in cumulative footprints, whereas values above zero indicate a reduction in cumulative footprints. ZAW: zero avoidable waste scenario, AMC: advanced material circulation, 3.5 °C: IMAGE SSP2-RCP6, and 1.5 °C: IMAGE SSP2-RCP19. Interest rates are applied to the cumulative EF.

Overall, waste management strategies and energy transition pathways influence the carbon, material, and economic footprints of CDW management in different ways. Transitioning to a near-100% recovery rate (ZAW scenario) only results in a 5–6%, 5%, and 10% additional reduction in cumulative carbon, material, and economic footprints, compared to BAU, respectively, across Europe. However, adopting advanced circularity strategies (AMC scenario) is projected to deliver more footprint reduction by 38–48%, 7–8%, and 25%, respectively, compared to the BAU scenario. Moreover, following the 1.5 °C energy transition pathway reduces the relative cumulative CF savings attributable to material circulation by 17–23%, depending on waste management scenarios, compared with the 3.5 °C pathway, as cleaner primary production narrows the emission gap between primary and secondary materials. In contrast, the effect of energy transition on MF is negligible, at approximately 1% across all scenarios. These results indicate that advanced CDW circularity systems are critical for maximizing carbon and economic benefits, while energy transitions may moderate the relative climate advantage of material recovery. Nevertheless, material recovery retains essential sustainability advantages beyond climate mitigation, including reduced virgin material extraction, lower MFs, and enhanced resource security.

### 3.4 Decarbonization and Economic Gains Primarily Driven by Metal Recycling

The four leading contributors to the decarbonization, dematerialization, and economic gains—Germany, the UK, France, and Italy—are central to Europe’s sustainable CDW management (Figure 4A). These four countries collectively account for 73–76% of cumulative decarbonization benefits, 71% of cumulative dematerialization efforts, and 75–75% of the cumulative economic gains, depending on scenarios (Figure 4C). Among the remaining countries, The Netherlands stands out, with 7–8% of cumulative decarbonization, 6% of cumulative dematerialization, and 8% of the cumulative economic gains. The rest of the countries are individually below 5% in all three footprints.

Material-wise, metal recycling is the primary driver of decarbonization, with 766–905 (25–36%) Mt CO_2_-eq of GHG reduction from recycling mixed metals, 572–939 (23–34%) Mt CO_2_-eq from iron and steel, 267–309 (9–13%) Mt CO_2_-eq from aluminum, and 167–249 (6–10%) of Mt CO_2_-eq from copper, depending on scenarios (Figure 4B). Together, these contributions account for approximately 67–89% of the cumulative CF reduction, depending on scenarios (Figure 4C). Recycling per unit of iron and steel saves much less CF (0.5–1 kg CO_2_-eq/kg) compared to aluminum (4–5 kg CO_2_-eq/kg) and copper (3–6 kg CO_2_-eq/kg). Yet, the large volume of iron and steel recycling results in a cumulative CF reduction 2–3 times greater than that of aluminum and 3–4 times that of copper. Adopting more advanced processing methods in the AMC scenarios—such as dismantling metal components for reuse—can achieve an additional 170–222 Mt CO_2_-eq of GHG mitigation compared to the BAU scenario.

It is important to note that downcycling waste concrete as backfill or low-quality aggregate leads to 74–89 Mt CO_2_-eq more GHG emissions compared to using natural sand and gravel, as observed in the BAU and ZAW scenarios (Figure 4A). In contrast, recycling concrete into high-quality secondary gravel, sand, and supplementary cementitious materials can reduce 132–346 Mt CO_2_-eq of cumulative CF. This reveals the untapped climate mitigation potential in stony minerals like concrete, given its large volumes processed. While metal recycling offers considerable decarbonization benefits, the impact of recycling concrete remains limited—averaging under 0.1 kg of CO_2_-eq saved per kg recycled. Therefore, even under an ambitious circular economy scenario (AMC), concrete circulation contributes only 5–10% of the cumulative decarbonization (Figure 4C).

Metal recycling likewise dominates economic value recovery within CDW management across Europe. The circular use of metals yields €642–655 billion in economic gains (Figure 4B), accounting for 77–95% of the total value derived from CDW management (Figure 4C). Compared to the BAU scenario, the ZAW strategy could provide an additional €2 billion in value, while the AMC scenario could yield €14 billion. However, concrete circulation only accounts for 3% of the value created at maximum.

### 3.5 Dematerialization Mainly Achieved through Concrete Recovery

Although negligible in decarbonization and economic gains, waste concrete circulation plays a dominant role in dematerialization. The recovery of concrete in the EU27 + 4 reduces the cumulative MF by approximately 14.0–14.8 Gt (Figure 4B), contributing to 57–60% of cumulative dematerialization, depending on scenarios (Figure 4C). Brick, as well as tile and ceramic, also make a considerable contribution, representing 7–9% and 6%, respectively. Collectively, mineral-based materials contribute around 72–74% of the cumulative dematerialization. While the role of metal recycling in dematerialization is smaller, it remains notable, constituting about 25–27% of the cumulative total.

### 3.6 Sensitivity to the Quantity of Waste Generation

The largest uncertainty in this study stems from waste generation quantities. A sensitivity analysis was conducted to evaluate how fluctuations in these quantities affect cumulative footprints. Given that minerals and metals are the primary contributors, the analysis includes five mineral waste fractions—concrete, brick, tile and ceramic, gypsum, and glass—and four metallic fractions—copper, aluminum, iron and steel, and mixed metals. For each fraction, a 10% increase in annual waste generation was simulated, and the resulting impacts on cumulative decarbonization, dematerialization, and economic gains are presented in Figure 5.

**Figure 5:**
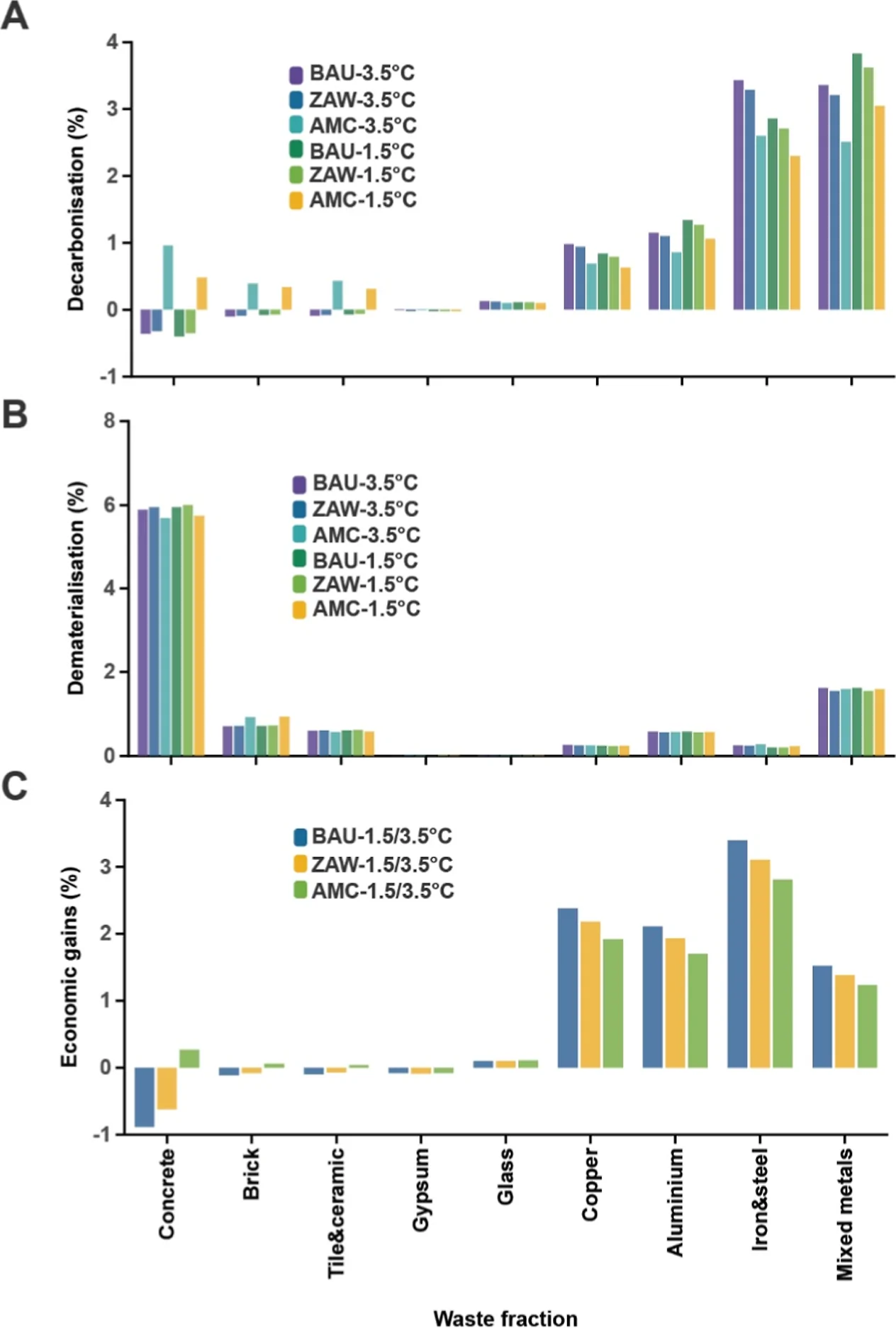
Sensitivity analysis of the effects of a 10% annual waste quantity increase on cumulative carbon, material, and economic footprints. (A) Effects of waste quantity increase on decarbonization. (B) Effects of waste quantity increase on dematerialization. (C) Effects of waste quantity increase on economic gains.

A 10% increase in CDW generation yields material-specific outcomes. Ferrous and mixed metals show the highest sensitivity: ∼1–4% improvements in cumulative decarbonization and economic gains. Copper and aluminum each deliver ∼1% CF reduction. Concrete, brick, tiles, and ceramics reduce carbon and economic footprints only under high-value circularity (AMC scenario, ∼1% CF reduction); under BAU or ZAW, they increase emissions and costs. Gypsum and glass show minimal sensitivity due to low volumes. Mineral and metal recovery consistently supports dematerialization; concrete (6%) and mixed metals (2%) drive the largest MF reductions. The findings underscore the need for material-specific, high-value circularity strategies to turn waste volume increases into climate and economic opportunities.

## 4 Discussion

### 4.1 Pathways for Future CDW Management in Europe

Overall, the results demonstrate that adopting circularity strategies for CDW management can effectively achieve comprehensive benefits in decarbonization, dematerialization, and economic gains across Europe. Key findings and future outlook include the following four points.

First, from a national perspective, Germany, the UK, France, and Italy play a dominant role, collectively contributing to over 70% of the cumulative decarbonization, dematerialization, and economic gains derived from CDW management in Europe. Each of these countries has the capacity to determine whether the EU’s 70% CDW recovery target could be achieved. This underscores the critical need for targeted policy support, technological innovation, and infrastructure investment in these high-impact nations to accelerate the advanced circularity transition and maximize region-wide circular economy benefits.

Second, different CDW fractions contribute asymmetrically to these outcomes: metals, which constitute only about 6% of CDW by mass in Europe, account for up to 90% of cumulative decarbonization and economic value due to their high embodied emissions in production and market price. In contrast, mineral fraction drives up to 70% of dematerialization benefits owing to their large volume and avoided natural resource extraction. To maximize systemic benefits, policy and investment strategies should be tailored to specific metallic and mineral streams, recognizing and balancing the inherent trade-offs among decarbonization, dematerialization, and economic value.

Third, enhancing CDW management in Europe requires a shift in focus from quantity-oriented recovery to quality-driven material circulation.^67^ The analysis shows that zero-waste approaches yield only marginal improvements under such conditions. Instead, transitioning to an advanced CDW management system that prioritizes reuse and recycling with quantifiable targets would significantly amplify decarbonization and economic outcomes. For high-impact metals, this means prioritizing advanced sorting and purification technologies to capture their exceptional decarbonization potential and market value. For voluminous mineral fractions, the focus should shift to avoiding landfills while exploring opportunities for producing high-grade recycled aggregates for structural use.

Finally, while energy transition pathways have a limited influence on the MF of CDW management, they hold substantial potential for decarbonizing material production processes. As primary production becomes cleaner—particularly for energy-intensive materials such as aluminum, steel, and cement—the relative carbon advantage of circularity strategies may diminish. To address this challenge, waste managers need to integrate renewable energy deployment and industrial CCS with improvements in recovery efficiency. Such a coordinated approach is essential to maintain the climate effectiveness and competitiveness of circular economy pathways.

### 4.2 Special Attention to Material Criticality

Metals play a multifaceted role in CDW management, combining not only high economic value and significant decarbonization potential but also strategic importance for key industries such as the renewable energy sector. This study includes the recycling of steel and iron, aluminum, and copper—materials for which the construction sector is the primary end-user in the EU, accounting for approximately 45%, 25%, and 50% of their total usage, respectively.^68^ Among these, aluminum and copper are prioritized under the European Critical Raw Materials Act (ECRA) due to their strategic importance.^69^

Beyond aluminum and copper, the construction industry relies on a wide array of critical raw materials (CRMs) and strategic raw materials (SRMs). For example, lithium, a critical element for traction batteries, is also used in concrete to prevent alkali–silica reaction, in tiles and ceramics to enhance strength, and in flat glass to improve durability.^70^ Although the exact extent of lithium use in concrete remains uncertain, a tentative estimate—assuming a concentration of 0.02% (Text S4.1, Supporting Information) by mass as a hardening accelerator—suggests that cumulative lithium embedded in waste concrete across the 31 European countries from 2010 to 2050 could reach ∼3 Mt, nearly one-tenth of global lithium reserves in 2024.^71^ The use of lithium in the ceramic and glass industry is more evident—with a range from <0.5% to 7.5% of Li_2_O required for ceramic manufacturing—accounting for approximately 14% of global lithium end-use in 2021.^70^

Furthermore, alloys need to integrate CRMs and SRMs to improve mechanical performance: tungsten, vanadium, titanium, and cobalt in steel; phosphorus in copper alloys; and vanadium, magnesium, bismuth, and titanium in aluminum alloys.^72^ Although the concentrations of these materials are low, the enormous scale of global construction implies substantial demand that may compete with other sectors. For instance, with nickel concentration in steel estimated at ∼0.1% (Text S4.2, Supporting Information), ∼1 Mt of nickel could be embedded in ferrous waste generated in the 31 European countries from 2010 to 2050—equivalent to more than one-quarter of global nickel production in 2024.^70^

The estimates in these two examples, while subject to considerable uncertainty, reveal an important reality: even materials present in low concentrations can represent a substantial—and often overlooked—consumption of CRMs and SRMs when scaled across the vast volumes of CDW generated in Europe. This underscores the urgent potential to strengthen circular economy practices in CDW management to improve CRM and SRM supply resilience. The ECRA mandates that by 2030, at least 25% of the EU’s annual consumption of CRMs and SRMs must be sourced from recycling.^69^ As of 2020, the EU had already achieved an impressive recycling rate of over 90% for ferrous, nonferrous, and mixed metals.^56^ Around 2015, recycled scrap met 36%, 29%, and 44% of the EU’s demand for steel, aluminum, and copper, respectively.^73^

However, effectively recovering tramp elements—residual metals such as copper, nickel, or chromium that accumulate in recycled steel—remains technically complex and often economically unviable with current technologies. While this challenge persists, it does not diminish the necessity of recycling metals. Comparable systematic and mandatory efforts should also be extended to mineral fractions, including concrete and ceramics. Downcycling these materials into low-value applications (e.g., backfilling) or disposing of them may lead to the irreversible loss of embedded CRMs and SRMs, thereby threatening future material supply security and undermining the transition to a low-carbon and circular transition.^1^

Building on the four key findings outlined above, the next subsection translates the identified pathways and material criticality considerations into concrete, actionable strategies for policymakers, industry stakeholders, and technology developers based on the comprehensive policy framework for advanced CDW management.^67^

### 4.3 Strategies for Operationalizing Circular CDW Management

#### 4.3.1 Establish a “Circular Economy Champions” Coalition among High-Impact Nations

Given the dominant role that Germany, the UK, France, and Italy play in EU CDW management, targeted intervention in these countries offers the highest systemic leverage. A dedicated “Circular Economy Champions” coalition should be established, supported by an EU-wide funding mechanism for cross-border infrastructure and innovation. Concrete actions include: (i) introducing binding national CDW circularity targets—such as reuse and recycling rates—that exceed the EU baseline; (ii) funding a joint R&D program focused on advanced sorting (e.g., AI-driven, sensor-based) and purification technologies for mixed CDW streams;^67^ and (iii) developing regional recovery hubs strategically located in these four countries to process high-value material flows from neighboring states.

### 4.4 Implement Material-Specific Circularity Targets and Quality-Driven Metrics

The asymmetric contributions of metals versus minerals demand tailored policy instruments that move beyond aggregate recovery rates. Past targets, such as the EU’s “70% CDW recovery by 2020”,^6^ drove progress but prioritized quantity over quality. Future policies should build on this by establishing material-specific recycling and reuse targets that differentiate between high-value applications and downcycling. Illustrative examples from the AMC scenario of this study include reaching a recycling rate of 20% for glass by 2040 and 80% for tiles and ceramics by 2050, as well as achieving a reuse rate of 13% for concrete and 29% for steel by 2050. The European Environment Agency aims to increase the circular material use rate—the ratio of secondary to total material consumption—to ∼22% by 2030.^74^ Material-specific targets could also be applied to the circular material use rate, particularly for CRMs and SRMs.

Moreover, to ensure the achievement of those targets, regulatory policies should be implemented: (i) mandating predemolition audits and a digital material passport to track material type, quantity, and quality, especially for high-value metal components such as copper wiring and steel beams; (ii) promoting designs for reuse and recycling to create the preconditions for advanced circularity; (iii) prohibiting the downcycling of high-grade metal scrap into lower-value applications without documented justification; (iv) setting minimum recycled content requirement for construction material in public procurement (e.g., 30% recycled aggregates for nonstructural applications and 15% for structural use with certification); (v) developing quality assurance protocols for reused and recycled material to overcome persistent market distrust. With such regulations in place, quality-driven, material-specific metrics would provide a more accurate and actionable framework for advancing circular CDW management than aggregate recovery targets alone.

#### 4.4.1 Decouple Circularity Value from Carbon Alone in the Context of Energy Transition

As primary production decarbonizes—particularly for energy-intensive materials such as aluminum, steel, and cement—the relative carbon advantage of circularity strategies may diminish. To maintain the competitiveness and climate effectiveness of circular economy pathways, recycling managers and policymakers must take coordinated action. Recommended actions include: (i) powering recycling facilities with on-site or contracted renewable energy (solar, wind, or biogas) to preserve or increase carbon savings from circularity as the grid decarbonizes; (ii) adopting real-time energy monitoring and optimization systems, such as AI-driven predictive control for crushers and sorters, to minimize operational emissions; and (iii) for energy-intensive fractions (aluminum, steel, cement recycling), exploring integration with point-source CCS where technically and economically feasible. On the policy side, circular economy indicators should be revised to include MF reduction and primary material substitution rates alongside carbon metrics, thereby reflecting the full value of circularity. A “carbon-adjusted circularity premium” could reward recycling pathways that achieve both high material substitution and low operational emissions, ensuring that circularity remains advantageous even as primary production becomes cleaner.

#### 4.4.2 Leverage Digital Traceability to Close Data Gaps and Enable Real-Time Monitoring

Finally, the systemic gaps in current waste statistics—unrecorded waste flows, soil distortion, and inadequate sectoral resolution—undermine evidence-based decision-making. The UK’s Mandatory Digital Waste Tracking (MDWT)^75^ system offers a replicable model: a unified digital platform that traces waste from generation through treatment and re-entry into markets as secondary raw materials. A similar interoperable system should be mandated across the EU via an update to the WFD, requiring all member states to implement digital platforms that track CDW from source to end-use, with a special focus on CRMs and SRMs. Scaling such digital traceability Europe-wide would close existing data gaps, enable near-real-time monitoring of material flows, significantly improve the accuracy and policy relevance of waste statistics, and thereby strengthen governance and accountability in circular CDW systems.

### 4.5 Limitations and Outlook

The robustness of this study is constrained by uncertainties in estimating CDW generation, waste management, and footprint modeling. Eurostat data sets provide the primary basis for waste generation and treatment, but their substance-oriented classification, inconsistencies in national reporting, and lack of details on individual CDW fractions introduce notable uncertainty.^43^ To address missing or aggregated flows, additional assumptions and extrapolations—such as using national statistics from the UK, Denmark, France, and Spain for estimating track ballast, characterizing metal composition, or applying sector-wide treatment practices to CDW fractions—were necessary, potentially biasing results. Moreover, Eurostat’s treatment statistics conflate recycling with downcycling, masking differences in material value retention, and discrepancies exist between reported and actual recycling rates (e.g., for nonferrous metals).

In footprint modeling, this study applied a substitution approach with a 1:1 displacement rule that assumes the produced 2RMs can fully replace equivalent quantities of corresponding primary materials in function and performance, thereby offsetting the associated environmental burdens and costs. This may overestimate the benefits of the 2RMs. The narrowing carbon advantage under cleaner energy scenarios is partly a methodological artifact of the substitution method; alternative allocation approaches would yield different results, and we acknowledge this dependency as a direction for future research. Then, this study used a process-based approach that underestimated the carbon and material footprints from the upstream and downstream supply chains. Furthermore, some primary production background processes (e.g., cement production) consider improvements in production efficiency and the application of CCS in the premise data set,^76^ whereas such advancements are largely overlooked in recovery-related foreground processes. This asymmetry may lead to an overestimation of the relative carbon benefits of recycling and other circularity strategies. Finally, although the interest rate is considered for economic assessment, the expenditure on the application of CCS technologies and material price fluctuations is not considered. A detailed description of these limitations is provided in Text S5, Supporting Information. Overall, these limitations highlight the need for more detailed, country-specific data, refined modeling assumptions, and dynamic assessments to improve the accuracy of evaluating CDW recovery pathways.

## Supplementary Material







## Notes

Source data for Figures 1–5 in the main text (XLSX)
EWC-stat-based CDW generation from Eurostat; estimated track ballast waste and asphalt waste; characterized CDW generation; CDW generation projection until 2050; treatment of concrete waste; treatment of brick waste; treatment of tile and ceramic waste treatment of insulation waste; treatment of gypsum waste; treatment of wood waste; treatment of glass waste; treatment of plastic waste; treatment of nonferrous metallic waste; treatment of ferrous waste; treatment of mixed metallic waste; and unitary carbon, material, and economic footprints for treatment per kg of waste (XLSX)
Detailed description of methods, including waste generation and projection, waste management, footprint modeling, critical mineral concentration calculation, limitation, waste project factors, waste management routes, inventory data, and footprint comparison (PDF)
