# Supplementary material for: Decarbonization, Dematerialization, and Economic Gains in Circular Economy for Construction and Demolition Waste Management in Europe
Source: Environ Sci Technol. 2026 Aug 30;60(36):25515–29. doi: 10.1021/acs.est.6c02401 (PMC13585050; doi:10.1021/acs.est.6c02401)
Supplement: Supplementary file 1 [file es6c02401_si_003.pdf]

Supporting Information for

Decarbonization, Dematerialization, and Economic Gains in Circular Economy for Construction and Demolition Waste Management in Europe

Chunbo Zhang, Jinxin Wang, Shuo Wang, Rupert J Myers, Julia A Stegemann

This file includes:

Text S1 to S5
Tables S1 to S4
Figures S1 to S4
References (1 to 64)

Other Supporting Materials for this manuscript include the following:

Source Data
Supporting Data

Text S1. CDW generation and projection

1.1 Waste generation statistics from Eurostat

The quantity of construction and demolition waste (CDW) generation is collected from Eurostat,[1] using a combination of the European Waste Classification for Statistics (EWC-Stat) codes[2] and the Statistical Classification of Economic Activities (NACE) codes[3]. The dataset covers the period 2010–2022 and includes waste data for EU27, Norway, Iceland, and the United Kingdom. Since 2020, Eurostat has ceased reporting waste data for the UK. To address the data gap in the UK, the missing values for 2020 and 2022 are extrapolated using the Waste Data Interrogator for England[4] and the waste statistics from the UK Department for Environment, Food & Rural Affairs[5]. In addition, CDW generation in Switzerland is estimated by applying the per-capita waste generation of the EU27[1] to its population data[6]. Eq. (1) illustrates the scope of the CDW generation data in a country from Eurostat. The Eurostat data on CDW generation in EU27+4 is provided in Sheet1 in Supplementary Data.

$$Q_{CDW}(t) = Q_{W061,F}(t) + Q_{W062,F}(t) + Q_{W063,F}(t) + Q_{W071,F}(t) + Q_{W074,F}(t) + Q_{W075,F}(t) + Q_{W077,F}(t) + Q_{W12B,F}(t) + Q_{W121,all}(t), \qquad t \in [2010,2022] \qquad (1),$$

where $Q_{CDW}(t)$ is the total CDW generation (including CDW from both buildings and infrastructure) in a given country in year t; $Q_{*,F}(t)$ is the generation of a specific waste category

* under EWC-Stat from the NACE construction sector (F); $Q_{*,all}(t)$ is the generation of the same waste category * across all NACE activities plus household.

1.2 Approximation of CDW from infrastructure

1.2.1 Approximation of track ballast. Eurostat reports rail line mileage statistics,[8] offering a potential pathway for estimation. Cyprus, Iceland, and Malta do not have any operational railways. Therefore, the quantity of track ballast in these three countries is not considered. Preliminary analysis shows that using linear regression based on rail mileage to calculate waste track ballast leads to significant overestimation. Therefore, waste track ballast is instead estimated as a percentage of total CDW. National statistics of Denmark (2011–2022),[9] France (2018),[10] Spain (2019–2022),[11] and the UK (2010–2022)[4] are used to derive the share of track ballast in CDW.

In the European List of Waste (LoW), track ballast is categorized as 17 05 07* (track ballast containing dangerous substances) and 17 05 08 (track ballast other than those mentioned in 17 05 07). While CDW is generally covered under Chapter 17, soils (17 05 03* and 17 05 04) and dredging spoils (17 05 05* and 17 05 06) are excluded. Waste generated from treating CDW is possibly included in Chapter 19[12] (with code 19 12 01, 19 12 02, 19 12 03, 19 12 04, 19 12 05, 19 12 07, and 19 12 09) is not considered either. Furthermore, waste entries with Recovery and Disposal (R&D) codes[13] R12, R13, D13, D14, and D15 are omitted to avoid double-counting when estimating both track ballast and CDW[14] in estimating both track ballast and CDW.

The calculated average share of track ballast in Denmark is 2.29% for the years 2011–2022, 0.09% in France for the year 2018, 0.01% in Spain for the years 2019–2022, and 1.92% in England for the time period 2010–2022 (Sheet2 in Supplementary Data). Based on these values, an overall average of 1.08% is derived and applied to other countries to estimate track ballast waste for the period 2010–2022. The estimated track ballast waste in EU27+4 for the period 2010–2022 is provided in Sheet2 in Supplementary Data.

1.2.2 Approximation of asphalt. Asphalt is related to the '17 03 bituminous mixtures, coal tars and tarred products' in the LoW.[15] The three major asphalt product types are asphalt-based paints, roofing asphalts, and paving asphalts. Among these, asphalt-based roofing materials can be recycled into paving asphalt by existing techniques,[16] which makes it hard to separate roofing asphalt from paving asphalt waste. Since asphalt-based paints and asphalt roofing materials represent a small fraction of asphalt compared to paving asphalt,[16] it is assumed that all asphalt waste originates from the road sector.

A preliminary attempt to estimate asphalt waste using linear regression based on paved road mileage showed a significant tendency to overestimate waste quantities. To address this, a percentile approach is adopted, calculating asphalt waste as a share of total CDW. The European Asphalt Pavement Association (EAPA) provides statistics for the production and reclamation of asphalt waste in some European countries.[17] Based on these data, the share of asphalt waste ranges from 5% in Hungary and Iceland to 48% in Sweden (Sheet2 in Supplementary Data). For countries with available data but missing years, missing values were extrapolated using the country's average share from 2013–2022. For countries with no data reported, an average value of 20% is

assumed. The estimated asphalt waste in EU27+4 for the period 2010–2022 is provided in Sheet2 in Supplementary Data.

1.3 Waste characterization

Before characterizing waste, track ballast and asphalt waste are removed from the mineral CDW (W12B and W121) to isolate mineral CDW from buildings, as shown in Eq. (2).

$$Q_{miernal}(t) = Q_{W12B,F}(t) + Q_{W121,all}(t) - Q_{track\ ballast}(t) - Q_{asphalt}(t),\ t \in [2010,2022] \quad (2),$$

where $Q_{miernal}(t)$ is the CDW generated from only buildings in a given country in year t; $Q_{W12B,F}(t)$ is the generation of a specific waste category W12B (Other mineral wastes) under EWC-Stat from the NACE construction sector (F) in a given country in year t; $Q_{W121,all}(t)$ is the generation of the same waste category W121 (Mineral waste from construction and demolition) across all NACE activities plus household in a given country in year t; $Q_{track\ ballast}(t)$ is the quantity of track ballast waste generated in a given country in year t; $Q_{asphalt}(t)$ is the quantity of asphalt waste generated in a given country in year t.

The EWC-Stat aggregate the 824 origin-based waste codes listed in the LoW[15] into 13 overarching, substance-oriented waste categories. Since the LoW system provides a more detailed and sector-specific system for the classification of CDW, the waste flows categorized under EWC-Stat codes are converted to LoW codes following the guidance provided by the Joint Research Centre (JRC)[18] and Eurostat[19] as follows:

- W121 Mineral wastes & W12B Other mineral waste: concrete (17 01 01), brick (17 01 02), tile and ceramic (17 01 03), mixed minerals (17 01 07), bitumen (17 03, subtracted), track ballast (17 05 07/08, subtracted), insulation (17 06), gypsum (17 08 02), and mixed CDW (17 09 04)
- W075 Wood wastes: wood (17 02 01)
- W071 Glass wastes: glass (17 02 02)
- W074 Plastic wastes: plastic (17 02 03)
- W061 Ferrous wastes: iron and steel (17 04 07)

- W062 Non-ferrous wastes: Al (17 04 02), Cu (17 04 01), Pb (17 04 03), Zn (17 04 04), Si (17 04 06), and cables (17 04 11)
- W063 Mixed metal wastes: mixed metals (17 04 07)
- W077 Waste containing Polychlorinated Biphenyls (PCB): CDW containing PCB (17 09 02)

Mineral wastes (W121 and W12B) are disaggregated based on the share of each mineral to CDW in each country in 2020 by the JRC.[18] Mixed minerals (17 01 07) and mixed CDW (17 09 04) are excluded from the analysis. Since asphalt is removed from CDW, mineral wastes (W121) are divided into concrete (17 01 01), brick (17 01 02), tile and ceramic (17 01 03), gypsum (17 08 02), and insulation (17 06).

Insulation in CDW consists of a mixture of mineral-based materials (e.g., stony wool and glass wool), organic-based materials (e.g., wood- and bamboo-based insulation), and polymers (e.g., expanded polystyrene and Polyurethane). Given its negligible overall volume in CDW, insulation is treated as part of the mineral fraction. To further disaggregate insulation, we adopt the European thermal insulation market shares in 2014:[20] 36% glass wool, 27% expanded polystyrene (EPS), 22% stone wool, 8% polyurethane (PU), 6% extruded polystyrene (XPS), and 1% others.

For non-ferrous wastes (W062), only aluminum and copper—the two dominant fractions in non-ferrous CDW—are considered. While JRC[21] reports their shares in CDW at the EU27 level with a high aluminum-to-copper ratio of 93:7, the share of aluminum appears too high. Therefore, national statistics are used to recalibrate the split: Denmark,[9] France,[10] Spain,[11] and the UK[4] report more balanced ratios, ranging from 51:49 (Denmark), 56:44 (France), 49:51 (Spain), to 58:42 (England). Based on these data, an average ratio of 58:42 is applied to allocate W062 non-ferrous wastes into aluminum and copper for the remaining countries.

The category mixed metals (W063) is not further disaggregated; however, its footprint is calculated using the weighted average shares of ferrous wastes (W061), aluminum, and copper—90:6:4. The characterized CDW generation quantities in EU27+4 from 2010 to 2022 are provided in Sheet3 in Supplementary Data.

1.4 Future waste projection

Future CDW generation in each investigated country is projected based on the growth rate relative to the base year 2020 (see Eq. (3)), as estimated through dynamic stock modelling of the building sector performed by JRC.[21] The projection runs from 2023 to 2050.

$$Q_w(t) = Q_w(2020) \times F_t,\ t \in [2023,2050] \qquad (3),$$

where $Q_w(t)$ is the quantity of a specific waste fraction *w* in year *t*; F_t is the growth factor of a specific waste fraction *w* generation in year *t*.

Since building retirement patterns vary across European regions, the pace of CDW generation also differs. Accordingly, the 31 investigated countries are grouped into four regions:

- Central and Eastern Europe: Bulgaria, Croatia, Czechia, Hungary, Poland, Romania, Slovakia, Slovenia
- Northern Europe: Denmark, Estonia, Finland, Iceland, Latvia, Lithuania, Norway, Sweden
- Southern Europe: Cyprus, Greece, Italy, Malta, Portugal, Spain
- Western Europe: Austria, Belgium, France, Germany, Ireland, Luxembourg, the Netherlands, Switzerland, the United Kingdom

The CDW growth factor by region is provided in Table S1. The panel dataset for prospective CDW generation in E27+4 is provided in Sheet 4 of the Supplementary Data.

Table S1 CDW growth factor in four regions of Europe

	Central and Eastern Europe	Northern Europe	Southern Europe	Western Europe
2020	100%	100%	100%	100%
2021	102%	104%	103%	103%
2022	106%	107%	106%	106%
2023	110%	110%	109%	109%
2024	113%	114%	113%	113%
2025	117%	118%	117%	116%
2026	121%	122%	121%	119%
2027	125%	126%	125%	123%
2028	129%	131%	129%	126%
2029	133%	136%	134%	130%
2030	137%	141%	139%	133%
2031	141%	146%	144%	137%
2032	146%	152%	149%	141%
2033	151%	157%	155%	144%
2034	156%	163%	161%	148%

2035	161%	170%	167%	152%
2036	167%	176%	173%	156%
2037	173%	183%	180%	160%
2038	179%	189%	187%	164%
2039	185%	196%	194%	168%
2040	192%	204%	201%	172%
2041	199%	211%	209%	176%
2042	207%	219%	217%	181%
2043	215%	226%	226%	185%
2044	223%	234%	235%	190%
2045	232%	242%	244%	194%
2046	241%	251%	253%	199%
2047	251%	259%	263%	204%
2048	261%	268%	273%	209%
2049	271%	277%	284%	213%
2050	283%	286%	295%	218%

Text S2. CDW Management

2.1 Historic CDW management

Data on historic CDW management (2010–2020) for each country are obtained from the Eurostat waste treatment dataset.[22] The historic treatment of concrete, brick, and tile/ceramic is derived from W121 Mineral waste from construction and demolition. At the EU level, concrete recycling is rarely performed due to high upfront costs.[23] For instance, in the Netherlands, only 3% of concrete was recycled in 2012.[24] Accordingly, it is assumed that all recovered concrete, bricks, and tiles/ceramics were managed through downcycling until 2020.

Eurostat reports that the recovery rate of glass waste exceeds 90% in most European countries (Eurostat, 2024c). However, only ~5% of recycled glass remains within the building sector.[25] In the Netherlands, ~8% of the flat glass was recycled in 2021, ~30% for the insulation industry, and ~50% for packaging.[26]

For gypsum, the EU-level recycling rate was 13% in 2013.[27] This rate is assumed to remain constant at 13% across all countries from 2010 to 2020.

For EPS/XPS, BASF reported treatment practices in Sweden, the Netherlands, Austria, Switzerland, Germany, Belgium, the UK, Czechia, Poland, France, Italy, and at the EU28+2 level

in 2018.[28] It is assumed that these reported countries maintained the 2018 treatment pattern throughout 2010–2020, while the EU28+2 pattern is applied to the remaining countries. The treatment of PU is assumed to follow the same pattern as EPS/XPS.

For glass wool and stone wool, treatment data are unavailable. Therefore, their treatment is assumed to follow that of insulation in general, based on national waste statistics in Denmark,[9] France,[10] Spain,[11] and the UK[4]: 2% recycled and 98% landfilled through the period of 2010 to 2020.

The treatment of wood, plastic, iron and steel, aluminum and copper, and mixed metals is modelled in line with the corresponding waste fractions: W075 Wood wastes, W074 Plastic wastes, W061 Ferrous wastes, W062 Non-ferrous wastes, and W063 Mixed metal wastes, respectively, based on the Eurostat waste treatment dataset for the period of 2010 to 2020.[22]

2.2 Prospective CDW Management

In the Business-As-Usual (BAU) scenario, treatment of each waste fraction remains the 2020 pattern till 2050. In the Zero Avoidable Waste (ZAW) scenario, the recoverability of each waste fraction is maximized by eliminating landfill and incineration by 2050. The Advanced Material Circulation (AMC) scenario aims not only for a 100% recovery rate but also emphasizes high-value-added waste management practices over backfilling and energy recovery, including reuse (all reuse operations in this study refer to ‘preparation for reuse’), upcycling, and recycling.

The maximum reusability and recyclability of the material derived from the report[18] and the study[29]. The upcycling potential of brick, tile and ceramic, and glass is modified based on Zhang et al.[29] The share of reuse, upcycling, and recycling for each material will linearly increase to their maximum levels by 2050. The detailed assumptions on the dynamic evolution of each treatment operation in three scenarios are explained in Table S2.

Table S2 CDW management routes from 2021 to 2050 under three scenarios. BAU: Business-As-Usual, ZAW: Zero Avoidable Waste, and AMC: Advanced Material Circulation.

Waste	BAU	ZAW	AMC
Concrete	Waste management practices remain	**Downcycling:** linearly increasing to 100% by 2050	**Reuse:** linearly increasing from 0% in 2030 to 13% by 2050

	constant at the 2020 level till 2050	**Landfill:** linearly phasing out by 2050	**Recycling:** linearly increasing from 0% in 2030 to 87% by 2050<br>**Downcycling:** linearly phasing out by 2050<br>**Landfill:** linearly phasing out by 2050
Brick	Waste management practices remain constant at the 2020 level till 2050	**Downcycling:** linearly increasing to 100% by 2050<br>**Landfill:** linearly phasing out by 2050	**Reuse:** linearly increasing from 0% in 2030 to 59% by 2050<br>**Upcycling:** linearly increasing from 0% in 2030 to 10% by 2050<br>**Recycling:** linearly increasing from 0% in 2030 to 31% by 2050<br>**Downcycling:** linearly phasing out by 2050<br>**Landfill:** linearly phasing out by 2050
Tile & ceramic	Waste management practices remain constant at the 2020 level till 2050	**Downcycling:** linearly increasing to 100% by 2050<br>**Landfill:** linearly phasing out by 2050	**Reuse:** linearly increasing from 0% in 2030 to 10% by 2050<br>**Upcycling:** linearly increasing from 0% in 2030 to 10% by 2050<br>**Recycling:** linearly increasing from 0% in 2030 to 80% by 2050<br>**Downcycling:** linearly phasing out by 2050<br>**Landfill:** linearly phasing out by 2050
Ferrous metal	Waste management practices remain constant at the 2020 level till 2050	**Downcycling:** linearly increasing to 100% by 2050<br>**Landfill:** linearly phasing out by 2050	**Reuse:** linearly increasing from 0% in 2030 to 29% by 2050<br>**Recycling:** linearly increasing to 100% by 2030; remaining a balancing share from 2030 onward<br>**Landfill:** linearly phasing out by 2030
Non-ferrous metal	Waste management practices remain	**Downcycling:** linearly increasing to 100% by 2050	**Reuse:** linearly increasing from 0% in 2030 to 50% by 2050

	constant at the 2020 level till 2050	**Landfill:** linearly phasing out by 2050	**Recycling:** linearly increasing to 100% by 2030; remaining a balancing share from 2030 onward<br>**Landfill:** linearly phasing out by 2030
Mixed metal	Waste management practices remain constant at the 2020 level till 2050	**Downcycling:** linearly increasing to 100% by 2050<br>**Landfill:** linearly phasing out by 2050	**Reuse:** linearly increasing from 0% in 2030 to 50% by 2050<br>**Recycling:** linearly increasing to 100% by 2030; remaining a balancing share from 2030 onward<br>**Landfill:** linearly phasing out by 2030
Glass	Waste management practices remain constant at the 2020 level till 2050	**Upcycling (foam aggregate):** remaining constant since 2021<br>**Recycling:** remaining constant at 5% till 2050<br>**Downcycling (packaging):** remaining constant at 55% till 2050<br>**Downcycling (backfill/road aggregate):** remaining a balancing share till 2050<br>**Landfill:** linearly phasing out by 2050	**Reuse:** linearly increasing from 0% in 2030 to 20% by 2050<br>**Upcycling (foam aggregate):** remaining constant since 2021<br>**Upcycling (supplementary cement material):** linearly increasing from 0% in 2030 to 5% by 2050<br>**Recycling**: linearly increasing from 5% to 20% by 2040, and remaining constant thereafter till 2050<br>**Downcycling (packaging):** remaining a balancing share till 2050<br>**Downcycling (backfill/road aggregate):** remaining zero till 2050<br>**Landfill:** linearly phasing out by 2050
Timber	Waste management practices remain constant at the 2020 level till 2050	**Recycling:** linearly increasing to 60% by 2050 (if <60% in 2020); remaining constant at the	**Reuse:** linearly increasing from 0% in 2030 to 25% by 2050

		2020 level (if >60% in 2020)<br>**Energy recovery:** remaining a balancing share till 2050<br>**Incineration:** linearly phasing out by 2050<br>**Landfill:** linearly phasing out by 2050	**Recycling:** remaining a balancing share till 2050<br>**Energy recovery:** linearly increasing or decreasing to 40% by 2050<br>**Incineration:** linearly phasing out by 2050<br>**Landfill:** linearly phasing out by 2050
Insulation: Stone/glass wool	Waste management practices remain constant at the 2020 level till 2050	**Recycling:** linearly increasing to 100% by 2050<br>**Landfill:** linearly phasing out by 2050	**Recycling:** linearly increasing to 100% by 2050<br>**Landfill:** linearly phasing out by 2050
Insulation: EPS/XPS/PU	Waste management practices remain constant at the 2020 level till 2050	**Recycling:** slowly increasing, extrapolated based on historic trend<br>**Energy recovery:** remaining a balancing share till 2050<br>**Landfill:** linearly phasing out by 2050	**Recycling:** linearly increasing to 45% by 2050<br>**Energy recovery:** remaining a balancing share till 2050<br>**Landfill:** linearly phasing out by 2050
Insulation: Other	Waste management practices remain constant at the 2020 level till 2050	**Landfill:** remaining constant at 100% till 2050	**Landfill:** remaining constant at 100% till 2050
Gypsum	Waste management practices remain constant at the 2020 level till 2050	**Recycling:** linearly increasing to 95% by 2050<br>**Landfill:** linearly decreasing to 5% by 2050	**Recycling:** linearly increasing to 95% by 2050<br>**Landfill:** linearly decreasing to 5% by 2050
Plastic	Waste management practices remain constant at the 2020 level till 2050	**Recycling:** linearly increasing to 90% by 2050<br>**Energy recovery:** remaining a balancing share till 2050<br>**Incineration:** linearly phasing out by 2050<br>**Landfill:** linearly phasing out by 2050	**Recycling:** linearly increasing to 90% by 2050<br>**Energy recovery:** remaining a balancing share till 2050<br>**Incineration:** linearly phasing out by 2050<br>**Landfill:** linearly phasing out by 2050

Detailed time-series data on the waste treatment under three scenarios are provided in Sheet5 to 15 in the Supplementary Data. The efficiency of recovery-related processes and substituted virgin raw materials is provided in Table S3.

Table S3 Data sources for environmental inventory modelling for different treatment routes of each material

Waste	Treatment	Reference	Recovery process efficiency	Raw material substitution	Remark
Concrete	Reuse	Damgaard et al.[21]	100%	Concrete component	Preparing for reuse of waste concrete component
	Recycling	Zhang et al. [30]	100%	Gravel: 56.5%; Sand:34.75 %; Cement (CEM II/A): 8.75%	Processing waste concrete through mobile advanced dry recovery (ADR) with heat-air classification system (HAS)[31] process for new concrete production
	Downcycling	Butera et al. [32]	100%	Gravel	Processing waste concrete into backfill for road
	Landfilling	Ecoinvent 3.9 [33]	Nada	Nada	Referring to unit process 'treatment of waste concrete, inert material landfill'
Brick	Reuse	Damgaard et al.[21]; Zhang et al.[29]	100%	Clay brick	Preparing for reuse of waste brick
	Upcycling	Fořt and Černý [34]	100%	Portland cement	Upcycling waste brick into supplementary cementitious material (SCM)
	Recycling	Fořt and Černý [34]	100%	Concrete block	Processing waste brick into concrete block
	Downcycling	Fořt and Černý [34]	100%	Gravel	Processing waste brick into backfill for road
	Landfilling	Ecoinvent 3.9 [33]	Nada	Nada	Referring to unit process 'treatment of inert waste, sanitary landfill'
Tile & ceramic	Reuse	JRC report [21]; Zhang et al.[29]	Nada	Nada	Preparing for reuse of waste tile and ceramic
	Upcycling	Fořt and Černý [34]	100%	Clak brick	Upcycling waste brick into supplementary cementitious material (SCM)
	Recycling	Fořt and Černý [34]	100%	Portland cement	Processing waste brick into concrete block
	Downcycling	Fořt and Černý [34]	100%	Concrete block	Processing waste brick into backfill for road

	Landfilling	Ecoinvent 3.9 [33]	100%	Gravel	Referring to unit process 'treatment of inert waste, sanitary landfill'
Glass	Reuse	JRC report [21]; Zhang et al. [29]	100%	Uncoated flat glass	Preparing for reuse of waste flat glass sheet
	Upcycling (SCM)	Zhang et al. [35]	100%	Cement (CEM II/A)	Processing waste glass into SCM for the concrete industry
	Upcycling (foam aggregate)	Blengini et al. [36]	100%	Foam glass	Processing waste glass into foam glass aggregate for the insulation industry
	Recycling	Caro et al. [37]	97.6%	Uncoated flat glass	Processing waste flat glass into new flat glass
	Downcycling (packaging)	Vossberg et al. [38]	98%	Packaging glass	Processing waste glass into packaging container
	Downcycling (backfill/road base aggregate)	Caro et al. [37]	97.6%	Gravel	Processing waste glass into aggregate
	Landfilling	ecoinvent 3.9 [33]	Nada	Nada	Referring to unit process 'Treatment of waste glass, sanitary landfill – GLO
Insulation: Stone wool	Recycling	Zhang et al. [35]	100%	Limestone powder	Processing waste stone wool into SCM.
	Landfilling	ecoinvent 3.9 [33]	Nada	Nada	Referring to unit process 'treatment of waste mineral wool, inert material landfill'
Insulation: Glass wool	Recycling	Zhang et al. [35]	100%	Limestone powder	Processing waste stone wool into SCM.
	Landfilling	ecoinvent 3.9 [33]	Nada	Nada	Referring to unit process 'treatment of waste mineral wool, inert material landfill'
Insulation: EPS/XPS	Recycling	Caro et al. [37]	65%	Polystyrene	Processing waste EPS/XPS into Polystyrene
	Energy recovery	Damgaard et al. [21]	55% (15% electricity; 40% heat)	Electricity and heat	Combusting waste EPS/XPS to generate power (Calorific value of EPS: 46 MJ/kg [39]; CO_2 emission from combustion: 3.3kg CO_2/kg [40])

	Incineration	Damgaard et al. [21]	Nada	Nada	Incinerating waste EPS/XPS (CO_2 emission from combustion: 3.3kg CO_2/kg [40])
	Landfilling	ecoinvent 3.9 [33]	Nada	Nada	Referring to unit process 'treatment of waste polystyrene, sanitary landfill'
Insulation: PU	Recycling	Marson et al. [41]	100%	Polyol	Processing waste PU into polyol
	Energy recovery	Damgaard et al. [21]	55% (15% electricity; 40% heat)	Electricity and heat	Combusting waste EPS/XPS to generate power (Calorific value of EPS is 29.3 MJ/kg; CO_2 emission from combustion: 2.9 kg CO_2/kg [42])
	Incineration	Damgaard et al. [21]	Nada	Nada	Incinerating waste PU (CO_2 emission from combustion: 2.9 kg CO_2/kg [42])
	Landfilling	ecoinvent 3.9 [33]	Nada	Nada	Referring to unit process 'treatment of waste polyurethane, sanitary landfill'
Insulation: Other	Landfilling	ecoinvent 3.9 [33]	Nada	Nada	Referring to unit process 'treatment of waste mineral wool, inert material landfill' for stone/glass wool (59%); 'treatment of waste polyurethane, sanitary landfill' for EPS/XPS (8%); 'treatment of waste polystyrene, sanitary landfill' for PU (33%)
Gypsum	Recycling	Suárez et al. [43]	95.2%	Gypsum	Processing waste gypsum into new gypsum
	Landfilling	ecoinvent 3.9 [33]	Nada	Nada	Referring to unit process 'treatment of waste gypsum, sanitary landfill'
Ferrous metal	reuse	Damgaard et al. [21]; Zhang et al. [29]	100%	Reinforcing steel	Preparing for reuse of reclaimed iron and steel components
	Recycling	Haupt et al. [44]; Zang et al [45]	90.7%	Unalloyed steel	Recycling waste steel into new steel
	Landfilling	ecoinvent 3.9 [33]	Nada	Nada	Referring to the unit process 'treatment of scrap steel, inert material landfill'

Non-ferrous metal: Aluminum	Reuse	Damgaard et al. [21]; Zhang et al. [29]	100%	Aluminum ingot	Preparing for reuse of waste aluminum component
	Recycling	Argerich and Holmberg [46]	78.4%	Aluminum ingot	Processing waste aluminum into aluminum ingot
	Landfilling	ecoinvent 3.9 [33]	Nada	Nada	Referring to unit process 'treatment of waste aluminum, sanitary landfill'
Non-ferrous metal: copper	Reuse	Damgaard et al. [21]; Zhang et al. [29]	100%	Copper ingot	Preparing for reuse of waste copper component
	Recycling	Zhang et al. [47];	83.3%	Copper ingot	Processing waste copper into copper ingot
	Landfilling	ecoinvent 3.9 [33]	Nada	Nada	Referring to unit process 'treatment of copper slag, residual material landfill'
Mixed metal	Reuse	Modified based on the treatment of ferrous and non-ferrous metals	100%	Unalloyed steel; aluminum ingot; copper ingot	Composition: ferrous metal, aluminum, and copper—90:6:4
	Recycling	Modified based on the treatment of ferrous and non-ferrous metals	Ferrous: 90.7%; Al: 78.4% Cu: 83.3%	Unalloyed steel; aluminum ingot; copper ingot	Composition: ferrous metal, aluminum, and copper—90:6:4
	Landfilling	ecoinvent 3.9 [33]	Nada	Nada	Composition: ferrous metal, aluminum, and copper—90:6:4
Plastic	Recycling	Caro et al. [37]	78.5%	Polyvinylchloride	Processing waste PVC into new PVC
	Energy recovery	Damgaard et al. [21]	55% (15% electricity; 40% heat)	Electricity and heat	Combusting waste PVC to generate power (Calorific value of EPS is 41 MJ/kg [48]; CO_2 emission from combustion: 2.9 kg CO_2/kg [42])
	Incineration	Damgaard et al. [21]	Nada	Nada	Incinerating waste PVC to generate power (CO_2 emission from

					combustion: 2.9 kg CO_2/kg [42])
	Landfilling	ecoinvent 3.9[33]	Nada	Nada	Referring to unit process 'treatment of waste plastic, mixture, sanitary landfill'
Timber	Reuse	Damgaard et al. [21]	100%	Uncoated particle board	Preparing for reuse of waste timber component (the carbon sink effect per kg of wood: –1.8 kg CO_2/kg [49])
	Recycling	Damgaard et al. [21]	77.3%	Uncoated particle board	Processing waste wood into particleboard (the carbon sink effect per kg of wood: –1.8 kg CO_2/kg [49])
	Energy recovery	Damgaard et al. [21]	20%	Electricity and heat	Combusting waste PVC to generate power (Energy content of wood waste: 9.5 MJ/kg [50]
	Incineration	Damgaard et al. [21]	Nada	Nada	Incinerating waste wood to generate power
	Landfilling	ecoinvent 3.9 [33]	Nada	Nada	Referring to unit process 'treatment of waste wood, untreated, sanitary landfill'
Waste containing PCB	Incineration	ecoinvent 3.9 [33]	Nada	Nada	Referring to unit process 'treatment of used capacitor, to hazardous waste incineration'

Text S3. Footprint modelling

3.1 Environmental inventory

The lifecycle-based carbon footprint (CF), material footprint (MF), and economic footprint (EF) are mainly modified based on our previous study.[29] The CF and MF focus on the GHG emissions and material use in waste treatment, including transport, recovery, and disposal processes, and avoided primary production. Transport includes waste transport, recovered material transport, mobile recovery plant transport, construction material/product transport, avoided primary mineral material transport, and avoided primary timber material transport. Distances for these activities are taken from the study.[29]

Biogenic carbon in wood waste is considered in CF modelling. In a previous study,[29] it was assumed that the biogenic carbon stored in the timber will eventually be released when the reused or recycled waste timber reaches its end-of-life. To differentiate wood component reuse and recycling waste wood into particleboard from combustion and incineration, the carbon sink effect of wood is considered—1.8 kg CO_2-eq/kg dry wood.[49]

In the Integrated Model to Assess Global Environment (IMAGE) model, Western and Central Europe have different patterns of energy transition.[51] In the footprint modelling, Western Europe includes: Austria, Belgium, Denmark, Finland, France, Germany, Greece, Iceland, Ireland, Italy, Luxembourg, Malta, the Netherlands, Norway, Portugal, Spain, Sweden, Switzerland, and the United Kingdom. Central Europe includes: Bulgaria, Croatia, Cyprus, Czechia, Estonia, Hungary, Latvia, Lithuania, Poland, Romania, Slovakia, and Slovenia.

3.2 Economic inventory

The economic inventory data are presented in Table S4. Steady-state costs are used, while the time value of money is considered. The cumulative EFs are discounted using the interest rates for the cost of borrowing for corporations from the European Central Bank.[52] The interest rate after 2025 is assumed to remain at the 2025 level till 2050. The unitary CF, MF, and EF for each waste treatment activity are provided in Sheet16 in the Supplementary Data.

Table S4 Data sources for economic inventory modelling for different treatment routes of each waste. Note: Since fees on landfilling and incineration are not available at EU level, only taxes for landfilling and incineration are considered.

Waste	Treatment	Cost (€/tonne)	Reference	Revenue (€/tonne)	Reference
Concrete	Reuse	2.66	Damgaard et al. [21]	137.76	Zhang et al. [31]
	Recycling	9.78	Zhang et al. [31]	19.53	Zhang et al. [31]
	Downcycling	5.81	Calculated based on Zhang et al. [31]	4.50	Zhang et al. [31]
	Landfilling	42.50	EEA [53]	/	/
Brick	Reuse	2.66	Same as concrete	83.00	Ecoinvent 3.4 [54]

	Upcycling	21.38	Modified from Fořt and Černý [34]	70.00	Zhang et al. [31]
	Recycling	13.85	Modified from Fořt and Černý [34]	10.20	Zhang et al. [31]
	Downcycling	5.81	Same as concrete	4.50	Same as concrete
	Landfilling	42.50	EEA [53]	/	/
Tile & ceramic	Reuse	2.66	Same as concrete	380.00	UK market information
	Upcycling	21.38	Modified from Fořt and Černý [34]	70.00	Zhang et al. [31]
	Recycling	13.85	Modified from Fořt and Černý [34]	10.20	Zhang et al. [31]
	Downcycling	5.81	Same as concrete	4.50	Same as concrete
	Landfilling	42.50	EEA [53]	/	/
Glass	Reuse	2.66	Same as concrete	700.00	Business Analyt IQ [55]
	Upcycling (SCM)	96.70	Zhang et al.[35]	70.00	Ecoinvent 3.4 [54]
	Upcycling (foam aggregate)	160.00	Iodice et al.[56]	620.00	Iodice et al.[56]
	Recycling	85.02	Caro et al.[37]	683.20	Business Analyt IQ [55]
	Downcycling (packaging)	30.00	Iodice et al.[56]	45.00	Iodice et al. [56]
	Downcycling (backfill/road base aggregate)	5.81	Same as concrete	4.50	Same as concrete
	Landfilling	42.50	EEA [53]	/	/
Insulation: Stone wool	Recycling	115.26	Zhang et al.[35]	77.00	Ecoinvent 3.4 [54]
	Landfilling	42.50	EEA [53]	/	/
Insulation: Glass wool	Recycling	115.26	Zhang et al.[35]	77.00	Ecoinvent 3.4 [54]
	Landfilling	42.50	EEA [53]	/	/
Insulation: EPS/XPS	Recycling	160.00	Iodice et al. [56]	620.00	Iodice et al. [56]
	Energy recovery	/	/	23.64	Damgaard et al. [21]
	Incineration	24.00	EEA [57]	/	/
	Landfilling	42.50	EEA [53]	/	/
Insulation: PU	Recycling	160.00	Iodice et al.[56]	1463.44	Ecoinvent 3.4 [54]
	Energy recovery	/	/	23.64	Damgaard et al. [21]
	Incineration	24.00	EEA [57]	/	/
	Landfilling	42.50	EEA [53]	/	/
Insulation: Other	Landfilling	42.50	EEA [53]	/	/
Gypsum	Recycling	150.28	Calculated based on Suárez et al. [43]	55.00	UK market information

	Landfilling	42.50	EEA [53]	/	/
Ferrous metal	reuse	2.66	Same as concrete	537.00	Ecoinvent 3.4 [54]
	Recycling	148.55	Damgaard et al. [21]	537.00	Ecoinvent 3.4 [54]
	Landfilling	42.50	EEA [53]	/	/
Non-ferrous metal: Aluminium	Reuse	2.66	Same as concrete	3670.00	Ecoinvent 3.4 [54]
	Recycling	148.55	Damgaard et al. [21]	3670.00	Ecoinvent 3.4 [54]
	Landfilling	42.50	EEA [53]	/	/
Non-ferrous metal: copper	Reuse	2.66	Same as concrete	5520.00	Ecoinvent 3.4 [54]
	Recycling	148.55	Damgaard et al. [21]	5520.00	Ecoinvent 3.4 [54]
	Landfilling	42.50	EEA [53]	/	/
Mixed metal	Reuse	2.66	Same as concrete	929.85	Calculated based on the composition
	Recycling	148.55	Damgaard et al. [21]	929.85	Calculated based on the composition
	Landfilling	42.50	EEA [53]	/	/
Plastic	Recycling	160	Iodice et al. [56]	680.00	Iodice et al. [56]
	Energy recovery	/	/	23.64	Damgaard et al. [21]
	Incineration	24.00	EEA [57]	/	/
	Landfilling	42.50	EEA [53]	/	/
Timber	Reuse	/	/	615.22	Damgaard et al. [21]
	Recycling	/	/	602.74	Damgaard et al. [21]
	Energy recovery	/	/	23.64	Damgaard et al. [21]
	Incineration	24.00	EEA [57]	/	/
	Landfilling	42.50	EEA [53]	/	/
Waste containing PCB	Incineration	24.00	EEA [57]	/	/

3.3 Footprint quantification

Eq. (4) shows how the material/carbon/economic footprint of CDW management in a country is calculated.

$$F(t) = \sum_{w} \sum_{f} Q_w(t) \times S_{w,f}(t) \times I_{w,f}(t)\,,\ t \in [2010{,}2050] \quad (4),$$

where $F(t)$ is the material/carbon/economic footprint of CDW management in a given country in year *t*; $Q_w(t)$ is the quantity of a waste fraction *w* in a given country in year t; $S_f(t)$ is the share of a waste fraction *w* allocated to fate (treatment route) f in a given country in year t; $I_{w,f}(t)$ is the unitary material/carbon/economic footprint indicator of a waste fraction *w* undergoing fate f in a given country in year *t*.

3.4 Regional footprint mapping

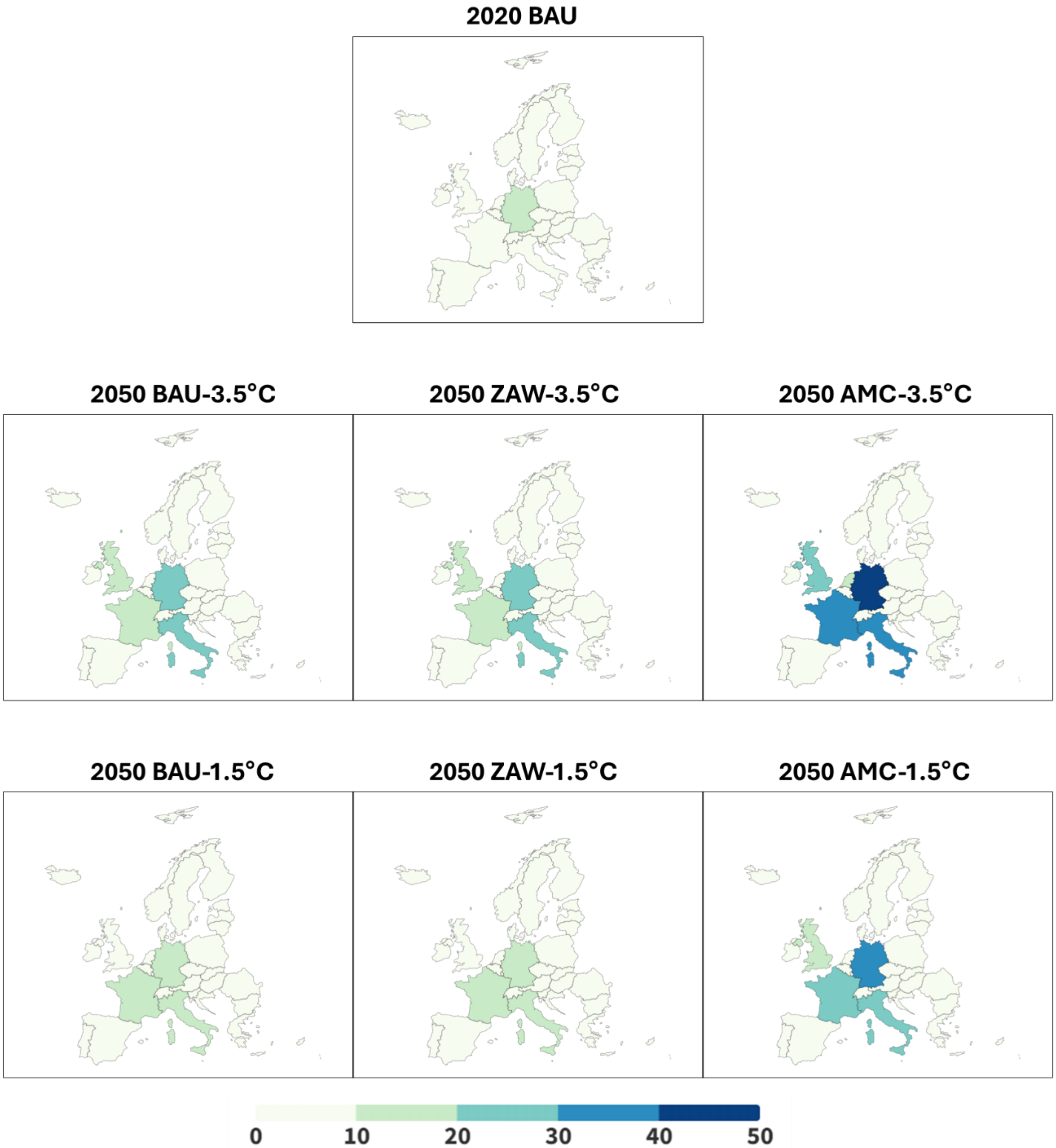

Figure S1 Comparison of carbon footprint reduction (unit: megatonne) in each scenario in 2020 and 2050

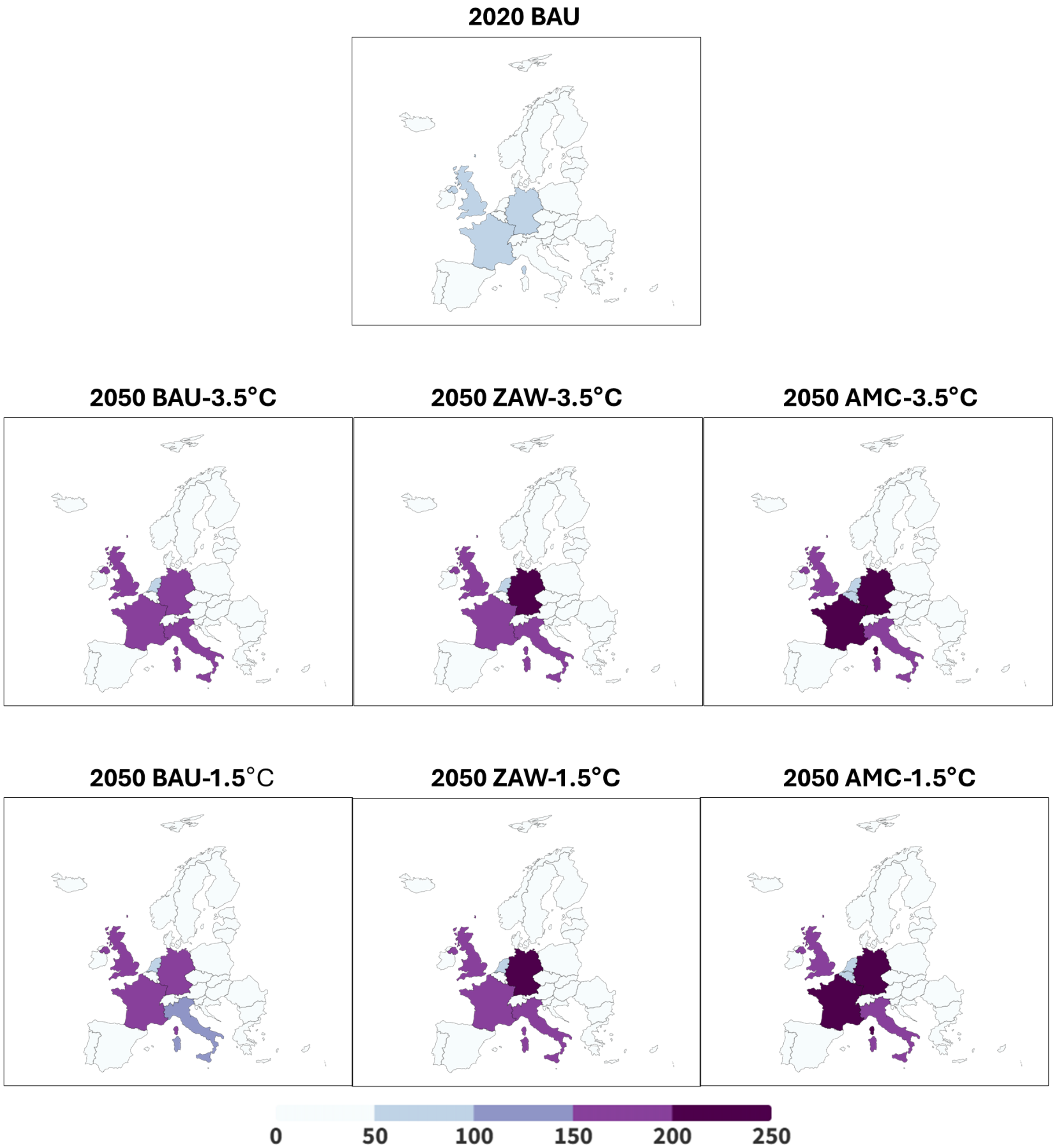

Figure S2 Comparison of material footprint reduction (unit: megatonne) in each scenario in 2020 and 2050

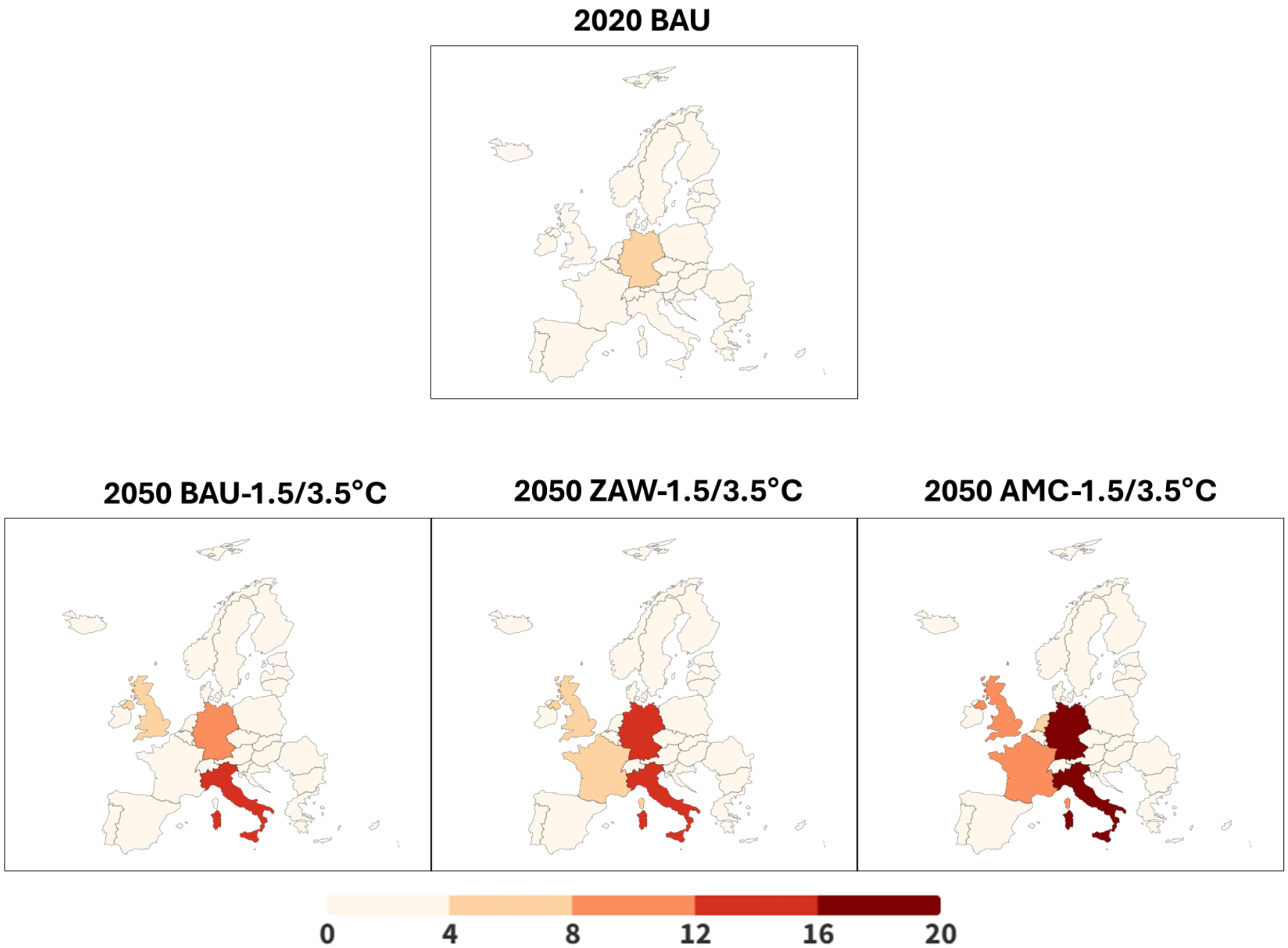

Figure S3 Comparison of economic footprint reduction (unit: € billion) in each scenario in 2020 and 2050

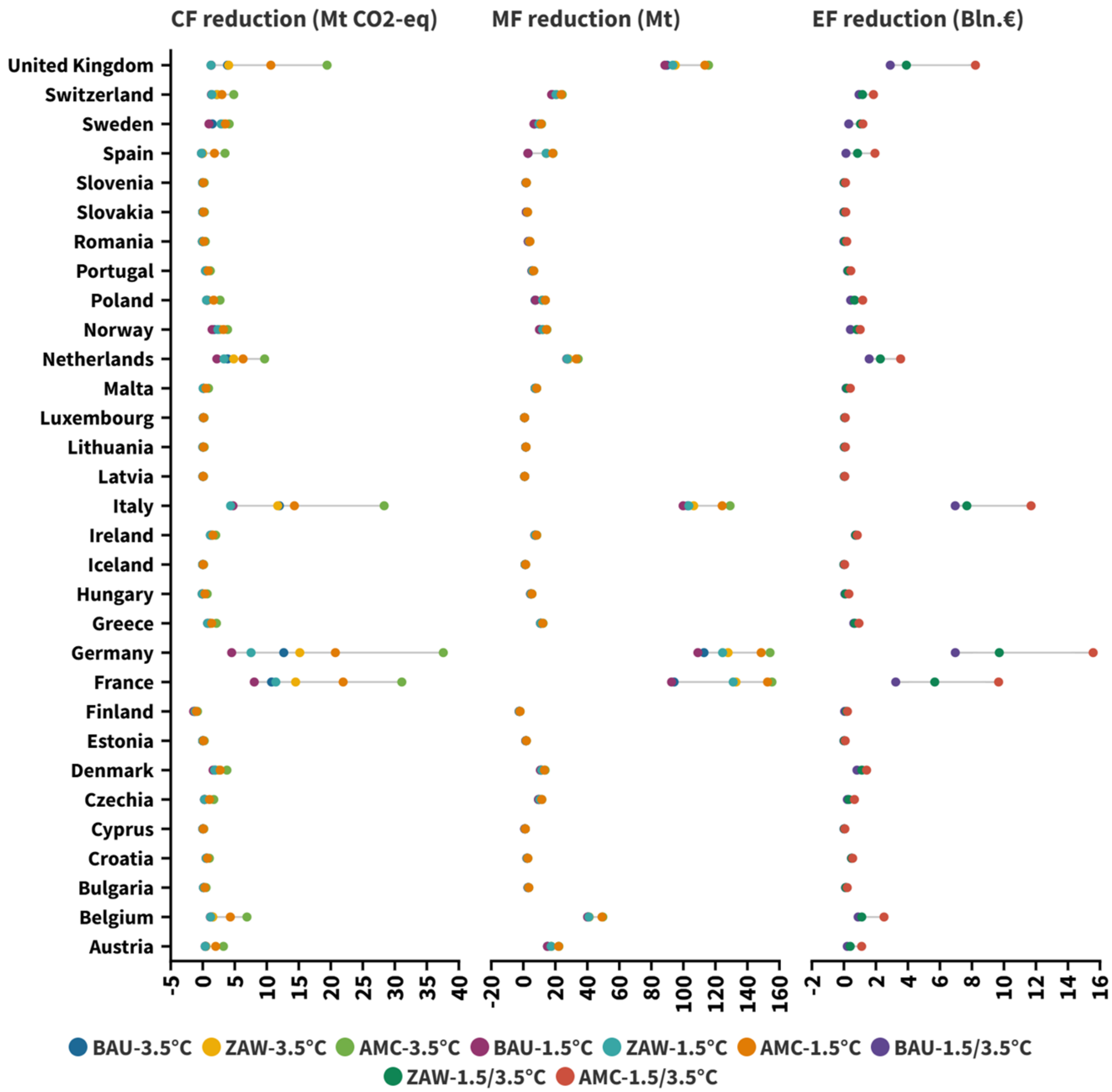

Figure S4 Difference in CF, MF, and EF in 2050 compared to 2020 levels (footprint of 2025 minus 2020) for each country. BAU: Business-as-Usual scenario, ZAW: Zero Avoidable Waste scenario, AMC: Advanced Material Circulation, 3.5°C: IMAGE SSP2-RCP6, and 1.5°C: IMAGE SSP2-RCP19.

Text S4. Concentrations of lithium and nickel

4.1 Lithium content in concrete

The benchmark concrete recipe from the VEEP project is: cement: 330 kg/m^3; sand: 1,020 kg/m^3; 950 kg/m^3 gravel; superplasticizer: 2.3 kg/m^3. The dry density of the concrete is 2,302 kg/m^3. Deng et al. used various dosages of Li_2SO_4 from 0.5–1.5% as a hardening accelerator in ordinary Portland cement paste.[58] Based on the molar mass, the mass fraction of lithium in Li_2SO_4 is 12.6% (13.88/109.94). Therefore, the estimated average lithium concentration in concrete is ~0.02%.

4.2 Nickel content in steel

The reported global crude steel production in 2021 was around 1,951 Mt.[59] The global nickel mining in 2021 was ~2.7 Mt,[60] and around ~1.9 Mt (69%) was used in the steel industry.[61] Therefore, the rough estimate of nickel concentration in steel is ~0.1%.

Text S5. Limitations

5.1 Waste generation

The limitations of this study mainly arise from uncertainties in estimating waste generation, waste management, and footprint modelling. Estimating the CDW generation is the first step of this study. The data for CDW quantity used in this study is derived from Eurostat's waste generation dataset.[1] However, that dataset neither directly provides the total CDW generation nor its composition. Eurostat employs a substance-oriented waste categorization system—EWC-Stat,[2] which complicates the separation of CDW from other waste streams. This introduces uncertainty in estimating the total CDW volume. In this study, an attempt was made to convert the EWC-Stat system into a sector-based classification system—the LoW, but the correspondence between these two systems remains unclear, adding further uncertainty to the composition and quantities of individual CDW fractions, adding further uncertainty to the composition and quantities of individual CDW fractions.

To facilitate this conversion, the UK's Waste Data Interrogator (WDI) was used to break down aggregated waste streams and estimate missing waste flows. For instance, CDW data from Eurostat includes transportation infrastructure waste (i.e., waste track ballast and asphalt), which has to be estimated individually and subtracted from the overall CDW. The proportion of track ballast in CDW from England was used to extrapolate this value for other countries, potentially leading to biased estimates.

Additionally, uncertainties around asphalt waste are even greater, as it is typically reclaimed through hot in-situ reprocessing. This makes it difficult to determine whether those activities should be reported as recycling, reuse, or refurbishment, or if the paving asphalt has become waste. Another challenge stems from the fact that Eurostat's dataset is based on national waste statistics. In some countries, such as the UK, Denmark, Germany, and the Netherlands, waste is reported based on collection rather than generation, which leads to underestimation as non-collected and exempted waste is excluded from the statistics.

Given the limitations in using Eurostat's waste statistics to estimate CDW generation, considerable uncertainties are inevitable. Future studies could improve accuracy by analyzing national waste statistics for each country and then aggregating them, or utilizing dynamic material flow analysis and waste intensity data to simulate CDW generation.

5.2 Waste management

Data on CDW management from 2010 to 2020 was sourced from Eurostat's waste treatment dataset.[22] Similar to the waste generation dataset, this dataset lacks sufficient detail to indicate the specific management routes of individual CDW streams, limiting its usefulness for decision-making related to advancing the level of waste management in the waste hierarchy. Certain simplifications were necessary for modelling CDW treatment in Europe. For instance, this study employed the management practices of "mineral waste from construction and demolition [W121]" to represent those of concrete, brick, and tile and ceramic from CDW. This study also used the waste management practices of waste (e.g., wood and plastic) from all sectors to represent those from the building sector.

Furthermore, the current classification of waste management operations does not distinguish between recycling and downcycling, which are often used interchangeably. For example, Eurostat reported that 78% of mineral CDW in EU27 was recycled in 2020,[22] while in practice, most waste concrete is still under unbound applications such as road bases, building foundations, and earthworks.[24] Therefore, in the BAU scenario of this study, it is assumed that all waste concrete is downcycled. The waste treatment dataset from Eurostat reports a near 100% average recycling rate for non-ferrous metals in Europe.[22] However, other sources suggest that copper specifically has a significantly lower recycling rate of around 70% in the EU.[62] Those discrepancies highlight the

challenges in accurately assessing the performance of waste management in different regions. Future research could focus on collecting more detailed data on CDW management practices in individual countries to provide a more accurate representation of CDW management across Europe.

5.3 Footprint modelling

In life cycle footprint modelling, recovery-based operations such as recycling and downcycling serve two primary functions: waste treatment and displacement of virgin materials. The majority of carbon and material footprint reduction stems from this displacement of primary materials. Several approaches exist for addressing multifunctionality, including system expansion, substitution, and allocation.[63] Previous studies have shown that different approaches may lead to similar conclusions, particularly in the case of concrete recycling.[30] In this study, the substitution approach was used to handle multifunctionality in waste recovery, with results expressed in negative carbon and material footprints. It is important to note that these calculated footprints do not represent actual quantities of carbon emissions or material use; rather, they aim to facilitate a systemic comparison between different scenarios.

In inventory modelling, the '1:1 displacement' rule was applied, which assumes that an equivalent unit of a secondary material can replace the same unit of a corresponding virgin material. Yet, the quality and economic value between secondary and virgin materials are not directly comparable—for instance, virgin cement versus supplementary cementitious materials.[30] Research indicates that using the "1:1 displacement" rule can lead to a substantial overestimation—around 40–60%—of the benefits provided by secondary materials.[64] Therefore, it is recommended that future studies incorporate a displacement rate to adjust the contribution of material recovery more accurately. This adjustment would account for the differences in quality and economic value between secondary and virgin materials, providing a more realistic assessment of the environmental benefits of recovery operations.

Additionally, this study considers the influence of clean energy transitions on the material recovery systems over time. However, it assumes that energy used for the production of primary materials remains at 2020 levels. Other than that, factoring in improvements in energy efficiency in both waste recovery and primary production processes is not considered either. The implementation of

green electricity and carbon capture and storage could significantly reduce the GHG emissions in carbon-intensive industries such as cement and steel. This may potentially diminish the advantages of circular economy strategies in construction material sectors. These evolving dynamics will be further explored in future research.

Supplementary References

(1) Eurostat. *Generation of waste by waste category, hazardousness and NACE Rev. 2 activity*. https://ec.europa.eu/eurostat/databrowser/view/env_wasgen/default/table?lang=en (accessed 2024-07-08).

(2) Eurostat. *Substance oriented waste statistical nomenclature (EWC-Stat Rev. 4)*. https://op.europa.eu/en/web/eu-vocabularies/dataset/-/resource?uri=http://publications.europa.eu/resource/dataset/ewc4#:~:text=Asset-,Substance oriented waste statistical nomenclature (EWC-Stat Rev.,4)&text=Description%3A,hazardous and non-hazardous waste (accessed 2024-06-30).

(3) EC. *List of NACE Codes*. https://ec.europa.eu/competition/mergers/cases/index/nace_all.html (accessed 2024-07-08).

(4) Defra. *2021 Waste Data Interrogator*. https://www.data.gov.uk/dataset/d8a12b93-03ef-4fbf-9a43-1ca7a054479c/2021-waste-data-interrogator (accessed 2023-04-13).

(5) Defra. *UK Statistics on Waste*. https://www.gov.uk/government/statistics/uk-waste-data/uk-statistics-on-waste#recovery-rate-from-non-hazardous-construction-and-demolition-cd-waste (accessed 2026-02-08).

(6) World Bank. *Population, total - Switzerland*. https://data.worldbank.org/indicator/SP.POP.TOTL?locations=CH (accessed 2025-09-04).

(7) Deloitte. *Construction and Demolition Waste Management in Finland*; 2015.

(8) Eurostat. *Total length of railway lines*. https://ec.europa.eu/eurostat/databrowser/view/TTR00003/default/table?lang=en&category=rail.rail_if (accessed 2024-08-25).

(9) Danish EPA. *Waste statistics 2020*. https://mst.dk/publikationer/2022/december/affaldsstatistik-2020.

(10) INERIS. *Industrial installations discharging pollutants (Installations industrielles rejetant des polluants)*. https://www.georisques.gouv.fr/donnees/bases-de-donnees/installations-industrielles-rejetant-des-polluants (accessed 2024-12-06).

(11) MITECO. *PRTR Waste Statistics*. https://prtr-es.es/informes/descargasResiduos.aspx (accessed 2024-12-06).

(12) EC. *Commision decision 2011/753/EU of 18 November 2011 establishing rules and calculation methods for verifying compliance with the targets set in Article 11(2) of Directive 2008/98/EC of the European Parliament and of the Council*. https://eur-lex.europa.eu/legal-content/EN/TXT/PDF/?uri=CELEX:32011D0753&from=EN (accessed 2020-05-22).

(13) EA. *D & R Code Flowchart*. https://assets.publishing.service.gov.uk/media/5a80caf7e5274a2e87dbbda3/DRAFT_Technical_Guidance_WM3.pdf.

(14) Zhang, C.; Noumbissié, L. T.; Zhang, J.; Zhang, C.; van Ewijk, S.; Stegemann, J. A. Collection and Reporting of Waste Data to Support Waste Management Policies. *npj Mater. Sustain.* **2026**, *4* (1), 6. https://doi.org/10.1038/s44296-025-00092-6.
(15) EC. *2001/118/EC: Commission Decision of 16 January 2001 amending Decision 2000/532/EC as regards the list of waste*. https://op.europa.eu/en/publication-detail/-/publication/429c2c5d-9be3-11e4-872e-01aa75ed71a1/language-en (accessed 2026-01-10).
(16) Arm, M.; Wik, O.; Engelse, C. J.; Erlandsson, M.; Sundqvist, J.-O.; Oberender, A.; Hjelmar, O.; Wahlström, M. *ENCORT-CDW: Evaluation of the European Recovery Target for Construction and Demolition Waste*; 2014. https://norden.diva-portal.org/smash/record.jsf?pid=diva2%3A724760&dswid=-1755.
(17) EAPA. *Asphalt in Figures*. https://eapa.org/asphalt-in-figures-archive/?utm_source=SOME&utm_medium=EAPA&utm_campaign=MARCH (accessed 2024-08-15).
(18) JRC. *Techno-Economic and Environmental Assessment of Construction and Demolition Waste Management in the European Union*; Luxembourg, 2024. https://doi.org/10.2760/721895.
(19) Eurostat. *Guidance on Classification of Waste According to EWC-Stat Categories*; 2010. https://ec.europa.eu/eurostat/documents/342366/351806/Guidance-on-EWCStat-categories-2010.pdf/0e7cd3fc-c05c-47a7-818f-1c2421e55604.
(20) Pavel, C. C.; Blagoeva, D. T. *Competitive Landscape of the EU's Insulation Materials Industry for Energy-Efficient Buildings*; Luxembourg, 2018. https://doi.org/10.2760/750646.
(21) JRC. *Background Data Collection and Life Cycle Assessment for Construction and Demolition Waste (CDW) Management*; Luxembourg, 2022. https://doi.org/10.2760/772724.
(22) Eurostat. *Treatment of waste by waste category, hazardousness and waste management operations*. https://ec.europa.eu/eurostat/databrowser/view/env_wastrt__custom_12571340/default/table?lang=en (accessed 2024-08-17).
(23) JRC. *Use of Recycled Aggregates in Concrete: Opportunities for Upscaling in Europe*; Luxembourg, 2023. https://publications.jrc.ec.europa.eu/repository/handle/JRC131294.
(24) Zhang, C.; Hu, M.; Yang, X.; Miranda-Xicotencatl, B.; Sprecher, B.; Di Maio, F.; Zhong, X.; Tukker, A. Upgrading Construction and Demolition Waste Management from Downcycling to Recycling in the Netherlands. *J. Clean. Prod.* **2020**, *266*, 121718. https://doi.org/10.1016/j.jclepro.2020.121718.
(25) Glass for Europe. *A Circular Economy Act to Support Flat Glass Closed-Loop Recycling Systems*; 2023. https://glassforeurope.com/a-circular-economy-act-to-support-flat-glass-closed-loop-recycling-systems/.
(26) Vlakglas Recycling Nederland. *Together we make glass infinite*. https://www.vlakglasrecycling.nl/index.php?page=home-en (accessed 2025-05-07).
(27) Jiménez Rivero, A.; Sathre, R.; García Navarro, J. Life Cycle Energy and Material Flow Implications of Gypsum Plasterboard Recycling in the European Union. *Resour. Conserv. Recycl.* **2016**, *108*, 171–181. https://doi.org/10.1016/j.resconrec.2016.01.014.
(28) BASF. *Waste Generation, Waste Streams and Recycling Potentials of HBCDcontaining EPS/XPS Waste in Europe and Forecast Model up to 2050*; Ludwigshafen, 2020.

https://www.psloop.eu/wp-content/uploads/2021/01/Conversio-study.pdf.
(29) Zhang, C.; Hu, M.; Sprecher, B.; Sacchi, R.; Yang, X.; Yang, S.; Verhagen, T. J.; Zhang, C.; Steubing, B.; Tukker, A. Revealing the Interplay between Decarbonisation, Circularity, and Cost-Effectiveness in Building Energy Renovation. *Nat. Commun.* **2025**, *16* (1), 7153. https://doi.org/10.1038/s41467-025-62442-1.
(30) Zhang, C.; Hu, M.; Meide, M. Van Der; Di, F.; Yang, X.; Gao, X.; Li, K.; Zhao, H.; Li, C. Life Cycle Assessment of Material Footprint in Recycling : A Case of Concrete Recycling. *Waste Manag.* **2023**, *155* (October 2022), 311–319. https://doi.org/10.1016/j.wasman.2022.10.035.
(31) Zhang, C.; Hu, M.; Dong, L.; Gebremariam, A.; Miranda-Xicotencatl, B.; Di Maio, F.; Tukker, A. Eco-Efficiency Assessment of Technological Innovations in High-Grade Concrete Recycling. *Resour. Conserv. Recycl.* **2019**, *149*, 649–663. https://doi.org/10.1016/j.resconrec.2019.06.023.
(32) Butera, S.; Christensen, T. H.; Astrup, T. F. Life Cycle Assessment of Construction and Demolition Waste Management. *Waste Manag.* **2015**, *44*, 196–205. https://doi.org/10.1016/j.wasman.2015.07.011.
(33) ecoinvent. *ecoinvent 3.9 cut-off database*. https://ecoinvent.org/.
(34) Fořt, J.; Černý, R. Transition to Circular Economy in the Construction Industry: Environmental Aspects of Waste Brick Recycling Scenarios. *Waste Manag.* **2020**, *118*, 510–520. https://doi.org/10.1016/j.wasman.2020.09.004.
(35) Zhang, C.; Hu, M.; Laclau, B.; Garnesson, T.; Yang, X.; Tukker, A. Energy-Carbon-Investment Payback Analysis of Prefabricated Envelope-Cladding System for Building Energy Renovation: Cases in Spain, the Netherlands, and Sweden. *Renew. Sustain. Energy Rev.* **2021**, *145*, 111077. https://doi.org/10.1016/j.rser.2021.111077.
(36) Blengini, G. A.; Busto, M.; Fantoni, M.; Fino, D. Eco-Efficient Waste Glass Recycling: Integrated Waste Management and Green Product Development through LCA. *Waste Manag.* **2012**, *32* (5), 1000–1008. https://doi.org/10.1016/j.wasman.2011.10.018.
(37) Caro, D.; Lodato, C.; Damgaard, A.; Cristóbal, J.; Foster, G.; Flachenecker, F.; Tonini, D. Environmental and Socio-Economic Effects of Construction and Demolition Waste Recycling in the European Union. *Sci. Total Environ.* **2024**, *908*, 168295. https://doi.org/10.1016/j.scitotenv.2023.168295.
(38) Vossberg, C.; Mason-Jones, K.; Cohen, B. An Energetic Life Cycle Assessment of C&D Waste and Container Glass Recycling in Cape Town, South Africa. *Resour. Conserv. Recycl.* **2014**, *88*, 39–49. https://doi.org/10.1016/j.resconrec.2014.04.009.
(39) Federation, B. P. *Expanded Polystyrene (EPS)*. https://www.bpf.co.uk/plastipedia/polymers/expanded-and-extruded-polystyrene-eps-xps.aspx#:~:text=The key benefit of using,gas at 48%2C000 kj%2Fkg.
(40) EPS Branchen. *100% Recyclable EPS - Expanded Polystyrene*; 2020.
(41) Marson, A.; Masiero, M.; Modesti, M.; Scipioni, A.; Manzardo, A. Life Cycle Assessment of Polyurethane Foams from Polyols Obtained through Chemical Recycling. *ACS Omega* **2021**, *6* (2), 1718–1724. https://doi.org/10.1021/acsomega.0c05844.
(42) QMRE. *Carbon Emissions and Plastic Waste*. https://www.qmre.ltd/carbon-emissions-and-plastic-waste/ (accessed 2025-09-01).
(43) Suárez, S.; Roca, X.; Gasso, S. Product-Specific Life Cycle Assessment of Recycled Gypsum as a Replacement for Natural Gypsum in Ordinary Portland Cement: Application to the Spanish Context. *J. Clean. Prod.* **2016**, *117*, 150–159.

https://doi.org/10.1016/j.jclepro.2016.01.044.
(44) Haupt, M.; Vadenbo, C.; Zeltner, C.; Hellweg, S. Influence of Input-Scrap Quality on the Environmental Impact of Secondary Steel Production. *J. Ind. Ecol.* **2017**, *21* (2), 391–401. https://doi.org/10.1111/jiec.12439.
(45) Zang, G.; Sun, P.; Elgowainy, A.; Bobba, P.; McMillan, C.; Ma, O.; Podkaminer, K.; Rustagi, N.; Melaina, M.; Koleva, M. Cost and Life Cycle Analysis for Deep CO2 Emissions Reduction of Steelmaking: Blast Furnace-Basic Oxygen Furnace and Electric Arc Furnace Technologies. *Int. J. Greenh. Gas Control* **2023**, *128*, 103958. https://doi.org/10.1016/j.ijggc.2023.103958.
(46) Argerich, J.; Holmberg, P. Life Cycle Assessment： A Comparison Between a New Produced and a Remanufactured Rear Subframe, Uppsala university, 2012.
(47) Zhang, W.; Li, Z.; Dong, S.; Qian, P.; Ye, S.; Hu, S.; Xia, B.; Wang, C. Analyzing the Environmental Impact of Copper-Based Mixed Waste Recycling-a LCA Case Study in China. *J. Clean. Prod.* **2021**, *284*, 125256. https://doi.org/10.1016/j.jclepro.2020.125256.
(48) Igniss Energy. *Calorific value (CV) of waste - "Waste to Energy."* https://www.igniss.com/calorific-value-waste (accessed 2025-09-01).
(49) Sustainability Impact Metrics. *Eco-costs calculations on wood*. https://www.ecocostsvalue.com/lca/wood-lca-issues/ (accessed 2025-09-01).
(50) Lu, H. (Ray); El Hanandeh, A. Life Cycle Assessment of ACQ-Treated Veneer Based Composite (VBC) Hollow Utility Poles from Hardwood Plantation Mid-Thinning. *Sustain. Prod. Consum.* **2016**, *5*, 36–50. https://doi.org/10.1016/j.spc.2015.11.002.
(51) PBL. *Integrated Model to Assess the Global Environment (IMAGE) 3.2*. https://models.pbl.nl/image/index.php/Welcome_to_IMAGE_3.2_Documentation.
(52) European Central Bank. *Cost of borrowing for corporations - euro area , Euro area (changing composition), Monthly*. 2025. https://data.ecb.europa.eu/data/datasets/MIR/MIR.M.U2.B.A2I.AM.R.A.2240.EUR.N (accessed 2025-09-01).
(53) EEA. *Overview of landfill taxes on municipal waste used in EU Member States, 2023*. https://www.eea.europa.eu/en/analysis/maps-and-charts/overview-of-landfill-taxes-on?activeTab=6fbd444d-c422-4a78-8492-fd496bd61b7a (accessed 2025-09-01).
(54) GreenDelta. *OpenLCA-based ecoinvent 3.4*. https://nexus.openlca.org/database/ecoinvent (accessed 2024-05-08).
(55) Business Analyt IQ. *Float glass price index*. https://businessanalytiq.com/procurementanalytics/index/glass-price-index/ (accessed 2025-01-01).
(56) Iodice, S.; Garbarino, E.; Cerreta, M.; Tonini, D. Sustainability Assessment of Construction and Demolition Waste Management Applied to an Italian Case. *Waste Manag.* **2021**, *128*, 83–98. https://doi.org/10.1016/j.wasman.2021.04.031.
(57) EEA. *Overview of taxes on the incineration of municipal waste used in EU Member States, 2023*. https://www.eea.europa.eu/en/analysis/maps-and-charts/overview-of-taxes-on-the?activeTab=6fbd444d-c422-4a78-8492-fd496bd61b7a (accessed 2025-09-01).
(58) Deng, Y.; Zhang, C.; Wei, X. Influence of Lithium Sulfate Addition on the Properties of Portland Cement Paste. *Constr. Build. Mater.* **2014**, *50*, 457–462. https://doi.org/10.1016/j.conbuildmat.2013.09.058.
(59) World Steel Association. *World Steel in Figures 2022*. https://doi.org/https://worldsteel.org/data/world-steel-in-figures/world-steel-in-figures-

2022/.
(60) USGS. *Minerals Yearbook - Metals and Minerals*. https://www.usgs.gov/centers/national-minerals-information-center/minerals-yearbook-metals-and-minerals (accessed 2025-09-01).
(61) Nickel Institute. *About Nickel*. https://nickelinstitute.org/en/nickel-applications/ (accessed 2025-09-01).
(62) EuRIC. *Metal Recycling Factsheet*; 2020. https://circulareconomy.europa.eu/platform/en/knowledge/metal-recycling-factsheet-euric.
(63) Guinée, J. B.; Heijungs, R.; Huppes, G.; Kleijn, R.; de Koning, A.; van Oers, L.; Wegener Sleeswijk, A.; Suh, S.; Udo de Haes, H. a.; de Bruijn, H.; van Duin, R.; Huijbregts, M. a. J.; Gorrée, M. *Handbook on Life Cycle Assessment: Operational Guide to the ISO Standards*; Dordrencht, 2001.
(64) Yang, J.; Duan, L.; Peng, S.; Heijungs, R.; Geng, X.; Wang, P.; Chen, W.; Yang, Y. Toward More Realistic Estimates of Product Displacement in Life Cycle Assessment. *Environ. Sci. Technol.* **2024**. https://doi.org/10.1021/acs.est.4c04006.
